# Exploring the mortality and cardiovascular outcomes with SGLT-2 inhibitors in patients with T2DM at dialysis commencement: a health global federated network analysis

**DOI:** 10.1186/s12933-024-02424-7

**Published:** 2024-09-03

**Authors:** Chung-An Wang, Li-Chun Lin, Jui-Yi Chen, Wei-Jie Wang, Vin-Cent Wu

**Affiliations:** 1 College of Medicine, Taipei Medical University, Taipei, Taiwan; 2 Department of Internal Medicine, National Taiwan University Hospital, 7 Chung-Shan South Road, Taipei, 100 Taiwan; 3 Division of Nephrology, Department of Internal Medicine, Chi-Mei Medical Center, Tainan, Taiwan; 4 Department of Health and Nutrition, Chia Nan University of Pharmacy and Science, Tainan, Taiwan; 5 Division of Nephrology, Department of Internal Medicine, Taoyuan General Hospital, Ministry of Healthy and Welfare, Taoyuan, Taiwan; 6 Department of Biomedical Engineering, Chung Yuan Christian University, Chungli, Taiwan; 7 Primary Aldosteronism Center of Internal Medicine, National Taiwan University Hospital, Taipei, Taiwan; 8 National Taiwan University Hospital Study Group of Acute Renal Failure (NSARF), Consortium for Acute Kidney Injury and Renal Diseases, Taipei, Taiwan; 9 Department of Internal Medicine, National Taiwan University Hospital, Room 1555, B4, Clinical Research Building, 7 Chung-Shan South Road, Taipei, 100 Taiwan

**Keywords:** Type 2 diabetes mellitus, Dialysis initiation, Major adverse cardiovascular events, Propensity score matching, Sodium-glucose cotransporter 2 inhibitors

## Abstract

**Background:**

Sodium-glucose cotransporter 2 inhibitors (SGLT-2is) have demonstrated associations with lowering cardiovascular outcomes in patients with type 2 diabetes mellitus (T2DM). However, the impact of SGLT-2is on individuals at dialysis commencement remains unclear. The aim of this real-world study is to study the association between SGLT-2is and outcomes in patients with T2DM at dialysis commencement.

**Methods:**

This is a retrospective cohort study of electronic health records (EHRs) of patients with T2DM from TriNetX Research Network database between January 1, 2012, and January 1, 2024. New-users using intention to treatment design was employed and propensity score matching was utilized to select the cohort. Clinical outcomes included major adverse cardiac events (MACE) and all-cause mortality. Safety outcomes using ICD-10 codes, ketoacidosis, urinary tract infection (UTI) or genital infection, dehydration, bone fracture, below-knee amputation, hypoglycemia, and achieving dialysis-free status at 90 days and 90-day readmission.

**Results:**

Of 49,762 patients with T2DM who initiated dialysis for evaluation, a mere 1.57% of patients utilized SGLT-2is within 3 months after dialysis. 771 SGLT-2i users (age 63.3 ± 12.3 years, male 65.1%) were matched with 771 non-users (age 63.1 ± 12.9 years, male 65.8%). After a median follow-up of 2.0 (IQR 0.3–3.9) years, SGLT-2i users were associated with a lower risk of MACE (adjusted Hazard Ratio [aHR] = 0.52, p value < 0.001), all-cause mortality (aHR = 0.49, *p* < 0.001). SGLT-2i users were more likely to become dialysis-free 90 days after the index date (aHR = 0.49, *p* < 0.001). No significant differences were observed in the incidence of ketoacidosis, UTI or genital infection, hypoglycemia, dehydration, bone fractures, below-knee amputations, or 90-day readmissions.

**Conclusions:**

Our findings indicated a lower incidence of all-cause mortality and MACE after long-term follow-up, along with a higher likelihood of achieving dialysis-free status at 90 days in SGLT-2i users. Importantly, they underscored the potential cardiovascular protection and safety of SGLT-2is use in T2DM patients at the onset of dialysis.

**Supplementary Information:**

The online version contains supplementary material available at 10.1186/s12933-024-02424-7.

## Introduction

 The prevalence of diabetes mellitus (DM) continues to surge on a global scale. Recent projections from the International Diabetes Federation (IDF) indicate that over half a billion individuals worldwide are currently grappling with diabetes, with expectations of a striking 46% escalation by 2045^1^. Notably, the percentage of incident ESRD patients caused by diabetes progressively increased from 22.1% in 2000 to 31.3% in 2015^2^. IDF also states that 30 to 40% of people living with diabetes develop CKD [3]. According to Kidney Disease Improving Global Outcomes (KDIGO), it has been estimated that 40% or more of people with diabetes will develop CKD, including a significant number who will develop kidney failure requiring dialysis or transplantation [4]. Given the well-established link between diabetes and the progression of chronic kidney disease (CKD), characterized by glomerular sclerosis, fibrosis, progressive albuminuria, and hypertension [5]. In addition to the challenges posed by diabetes-related long-term cardiovascular disease (CVD), individuals undergoing renal replacement therapy find themselves confronting an array of CVD [6, 7].

The introduction of sodium-glucose cotransporter 2 inhibitors (SGLT-2is), a novel class of oral antidiabetic drugs (OADs), has caused a paradigm shift in the treatment strategies for patients with type 2 diabetes mellitus (T2DM). These drugs have demonstrated the capacity to reduce major adverse cardiovascular events (MACE), heart failure hospitalization, and renal protection in patients with T2DM with established cardiovascular disease or those at risk [8–10]. Additionally, clinical trials have shown their benefits in patients with heart failure across the ejection fraction spectrum, regardless of the presence or absence of diabetes [11–13]. Trials specifically targeting patients with CKD have similarly shown cardio-renal protective effects. EMPA-KIDNEY trial and post-hoc analysis shows that SGLT-2is offer kidney benefits in CKD patients, irrespective of diabetes status, and even in those with estimated glomerular filtration rate (eGFR) < 20 mL/min/1.73 m²^14,15^. The update from the Kidney Disease Improving Global Outcomes (KDIGO) guideline now recommends the initiation of SGLT-2is at a lower eGFR threshold, reducing it from 25 to 20 mL/min/1.73 m², and suggests the continuation of these medications until the need for dialysis and the UK Kidney Association Clinical Practice Guideline recommends initiation of SGLT-2is in people with an eGFR below 20 mL/min/1.73 m² to slow progression of kidney disease [4, 16]. This significant change in recommendations signifies potential advantages for individuals with diabetes who require dialysis. As of May 2023, the U.S. FDA has revised its guidance on dapagliflozin by eliminating the previous contraindication related to patients undergoing dialysis [17]. Intriguingly, a similar pattern has emerged in Europe, with the European Medical Agency (EMA) not listing dialysis as a contraindication for dapagliflozin [18]. An exploratory analysis of DAPA-CKD trial indicated no significant safety concerns in dialysis patients [19]. To address this notable gap in the literature, our study endeavors to investigate the potential association between SGLT-2is and all-cause mortality and cardiovascular outcomes in patients with T2DM who initiated dialysis, compared to those initializing dialysis but are not receiving SGLT-2is.

## Methods

### Data sources

In this retrospective study, we leveraged the TriNetX Research Network, a global federated health research platform, and the data in the TriNetX Research Network is sourced from healthcare organizations (HCOs) [20]. The data set encompassed a broad spectrum of information, including patient demographics, diagnoses (aligned with the International Classification of Diseases, Ninth Revision, Clinical Modification [ICD-9-CM] and the International Classification of Diseases, Tenth Revision, Clinical Modification [ICD-10-CM] codes), procedures (documented with ICD-9-CM, the International Classification of Diseases, Tenth Revision, Procedure Coding System [ICD-10-PCS], and Current Procedural Terminology [CPT] codes), medications (coded according to the Veterans Affairs National Formulary and RxNorm ingredients), laboratory tests (categorized by LOINC), and healthcare utilization records from multiple HCOs, including hospitals, primary care units, and specialized facilities.

We utilized the TriNetX Research Network database, which includes EHRs of over 100 million patients across 93 healthcare organizations (HCOs) in five countries: Taiwan, Georgia, Colombia, Brazil, and the United States [21–27]. Patient-level data were analyzed using the built-in statistical tool on the TriNetX platform, based on Java (version 11.0.16), R (version 4.0.2, with packages Hmisc and Survival), and Python (version 3.7, with libraries lifelines, matplotlib, numpy, pandas, scipy, and statsmodels). The results were presented to investigators in an aggregated format. Further details about the database are available online and in previously published descriptions [28, 29].

This study using the TriNetX database obtained ethical approval from the Institutional Review Board of Chi-Mei Hospital, Tainan, Taiwan (No: 11202-002), and the institutional review boards of all participating hospitals. A waiver of informed consent was granted by the Western Institutional Review Board because this study was conducted using only aggregated statistical summaries of de-identified information. The study was conducted in accordance with the principles outlined in the Declaration of Helsinki [30] and adhered to the Strengthening the Reporting of Observational Studies in Epidemiology (STROBE) reporting guideline for its design.

### Study population

**Fig. 1:**
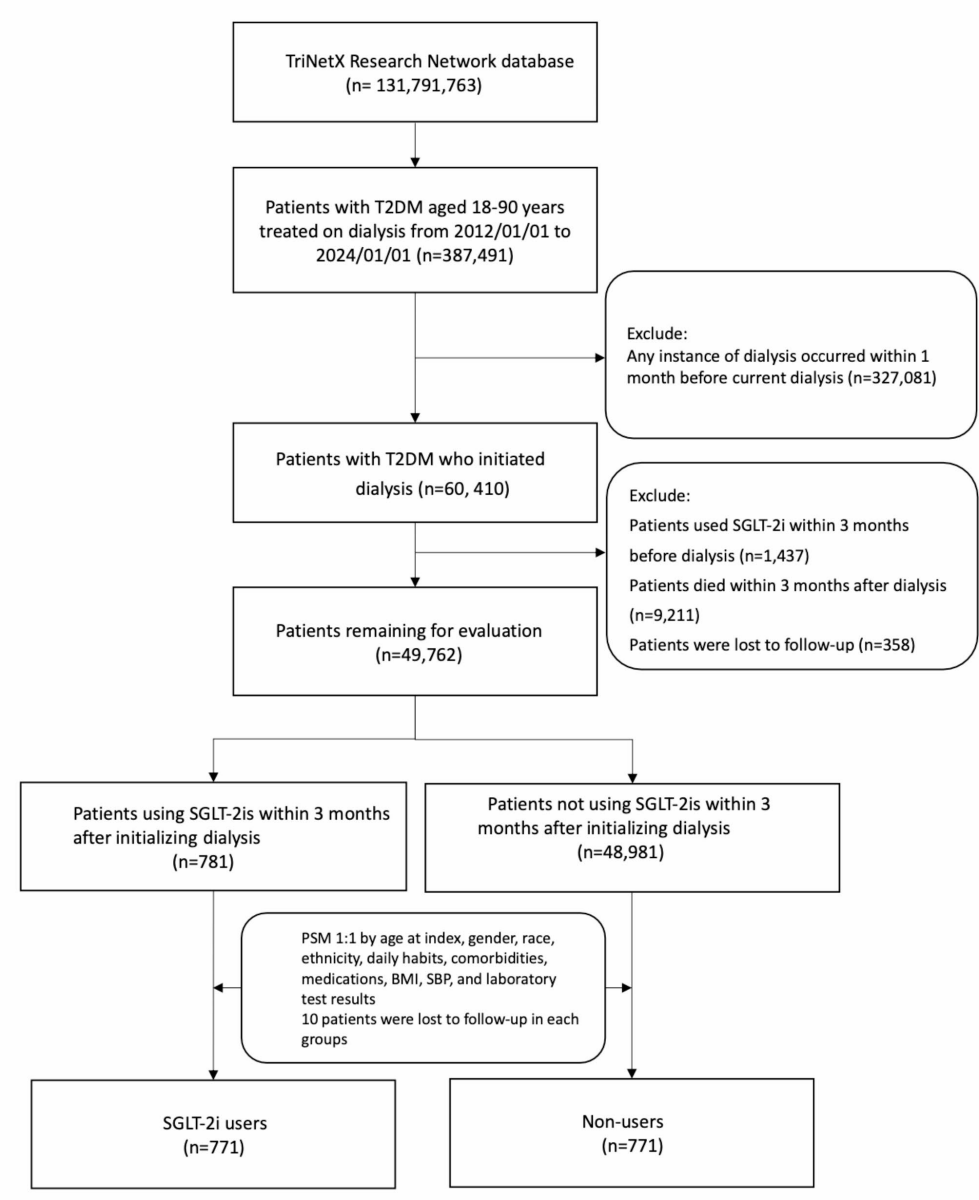
Enrollment algorithm for patients. *BMI* body mass index, *T2DM* type 2 diabetes mellitus, *PSM* propensity score matching, *SBP* systolic blood pressure, *SGLT-2is* sodium–glucose cotransporter 2 inhibitors

In this study, a cohort was established by selecting and organizing participants from the database, covering the period from January 1, 2012, to January 1, 2024, involving 131,791,763 individuals. The study included patients aged 18 to 90 years with T2DM who initiated dialysis during this period. Patients were categorized as SGLT-2i users if they had received a prescription for an SGLT-2is within 3 months following the commencement of dialysis. Patients were categorized as SGLT-2i users if they received a prescription for an SGLT-2is within 3 months of commencing dialysis. T2DM patients who did not use SGLT-2is during the specified period were grouped as controls, in line with our intention-to-treat (ITT) design. Exclusion criteria included any instance of dialysis within 30 days before the current dialysis session, and individuals who used SGLT-2is before initial dialysis for 3 months and passed away within 3 months following their initial dialysis. These criteria aimed to identify patients with T2DM undergoing acute dialysis or newly entering chronic dialysis treatment and a new-users design also implemented to ensure SGLT-2is who did not have a prior history of using SGLT-2is. The index date was set at 90 days after dialysis commencement. To address potential sources of protopathic or ascertainment bias [31], any occurrences of outcomes that transpired before the index date were meticulously excluded from the analysis. Enrollment algorithm of participants was illustrated in Fig. 1.

### Outcomes

The primary outcomes centered on two critical aspects of patient health: all-cause mortality and the occurrence of major adverse cardiovascular events (MACEs) during the follow-up period. These MACEs, representing a composite outcome, encompassed non-fatal myocardial infarction, non-fatal ischemic stroke, cardiovascular death/mortality, and hospitalization for unstable angina. Secondary outcomes included 3-Point Major Adverse Cardiovascular Events (3p-MACEs), which comprised non-fatal myocardial infarction, non-fatal ischemic stroke, and cardiovascular death/mortality. Additionally, the analysis extended to other safety outcomes and side effects such as ketoacidosis, UTI or genital infection, dehydration, bone fracture, below-knee amputation, hypoglycemia, dialysis-free status at 90 days, and 90-day readmission. Patients were followed until death, the last recorded entry in their health record, the completion of 5 years of follow-up, which starting after the index date, or until July 23, 2024, whichever occurred first. The detailed diagnostic, visit, and procedural codes used to define the outcomes can be found in the Supplementary Methods. To mitigate the potential impact of protopathic or ascertainment biases, we meticulously excluded any occurrences of secondary outcomes before the index date [22].

### Covariates

To account for differences in baseline characteristics between the two study groups, we incorporated specific covariate factors and potential confounding factors into our analysis. These factors included demographic variables such as age, gender, ethnicity, and race. Additionally, our analysis encompassed the evaluation of various comorbidities, including ever hospitalization within the past year, hypertensive diseases, peripheral vascular diseases, ischemic heart diseases, cerebrovascular diseases, chronic obstructive pulmonary diseases, asthma, chronic kidney disease (CKD), peritoneal dialysis, dementia, sleep disorders, depression, and neoplasms. We also considered clinical measures including body mass index (BMI), and systolic blood pressure and laboratory results such as, hemoglobin A1c, estimated glomerular filtration rate (eGFR), total cholesterol, alanine aminotransferase (ALT), B-type natriuretic peptide (BNP), potassium levels, and urine protein. Daily behaviors and habits, such as smoking and alcohol consumption and medication history were included, covering insulin, thiazolidinediones, glucagon like peptide-1-receptor (GLP-1) analogues, dipeptidyl peptidase-4 (DPP-4) inhibitors, sulfonylureas, aspirin, clopidogrel, statins, allopurinol, febuxostat, alpha-blockers, beta-blockers, calcium channel blockers (CCB), and the use of angiotensin-converting enzyme inhibitors (ACEI) or angiotensin II receptor blockers (ARB).

These variables were integrated into our analysis to adjust for any variations in the baseline characteristics of the study cohorts. To ensure the accuracy of our analysis and minimize multicollinearity, we utilized quantifiable continuous variables such as body mass index (BMI) and estimated glomerular filtration rate (eGFR), categorized appropriately, instead of relying solely on categorical variables like obesity and CKD. The detailed codes used to define the covariates can be found in the Supplementary Methods.

### Subgroup and sensitivity analysis

In our study, we were conducting a comprehensive subgroup analysis to explore potential variations across different subgroups. Patients were stratified according to age (≥ 65 or < 65 years), baseline estimated glomerular filtration rate (eGFR) (≥ or < 30 mL/min/1.73 m²), urine protein/creatinine ratio (UPCR) (≥ or < 300 mg/g), body mass index (BMI) (≥ or < 30 kg/m²), and HbA1c levels (≥ or < 7%). We also assessed outcomes based on the use of beta blockers, ACEI/ARB, the enrolled period (before 2018 or after 2018), the presence of cardiovascular disease (CVD), smoking history, the various types of SGLT-2is and advanced CKD (≥ or < 15 mL/min/1.73 m²).

### Positive and negative controls

To assess the reliability of our analytical approach and avoid systemic bias, we conducted negative outcome controls, including the incidence of skin cancer, herniated disc, hemorrhoids, COPD, URIs, and GERD, which based on prior knowledge or expectations [32, 33]. We selected Angiotensin II Receptor Blockers (ARB) as our positive exposure control, based on literature suggesting that ARB usage is linked to a reduced risk of all-cause mortality and MACE [34–36]. For our negative exposure control, we chose Histamine Type 2 Receptor Antagonists (H2 blockers) and antidepressants (Selective Serotonin Reuptake Inhibitors, SSRI) [37, 38].

### Landmark analysis for selection period and followed-up period

To address the impact of immortal or ascertainment bias, our series of landmark analyses involved initiating the follow-up period on the 14th, 30th, or 60th days post-acute dialysis. We also performed analyses across follow-up periods of 1, 2, 3, 4, and 5 years and compared these results to the overall study period. To ensure the robustness, we evaluated various exclusion criteria, including patients who died after dialysis initiation, and applied Cox regression model with different covariates. Additionally, we assessed the impact of SGLT-2is discontinuation timing by comparing continued use versus discontinuation within 3 months after the index date.

### Statistical analysis

Baseline characteristics of the SGLT-2i users and non-users groups were presented numerically as mean [SD] for continuous variables and as count and percentage for categorical variables. Categorical variables were compared using chi-squared tests, and continuous variables were compared using independent 2-sample t-tests. One-to-one PSM was performed using logistic regression and greedy nearest neighbor matching based on several factors, with a caliper of 0.1 pooled standard deviations to balance baseline characteristics between the two groups. Variables were considered adequately matched if the between-group difference was below 0.1, indicating a small difference [39]. Survival probabilities were estimated using the Kaplan-Meier method. Patients were censored on the day they received a kidney transplant or after the last recorded event if that event occurred within the time window of the study. Adjusted hazard ratios (aHRs) with 95% confidence intervals (CIs) were calculated using Cox proportional hazards regression models, while relative risks (RRs), odds ratios (ORs), and risk differences were also assessed.

Additionally, *E* values were used to provide insights into the potential impact of unmeasured confounders on the observed associations [40]. Missing data were addressed by excluding the respective cases to ensure complete datasets and maintain result integrity. Patients lost to follow-up were also excluded to minimize bias and inaccuracies due to incomplete data.

We further conducted a Bayesian analysis to update the probability of mortality based on the treatment. The prior probability of receiving treatment was set at 50%, with subsequent calculations using Bayes’ theorem to determine the posterior probability of mortality associated with SGLT-2is, which indicated a reduced risk of mortality compared to the baseline.

All statistical analyses were conducted using the analytic tool on the TriNetX platform and R, version 4.2.2. Statistical software SAS, version 9.2 (SAS Institute Inc), and Stata/MP software, version 16 (StataCorp LLC), were also used for data analysis. Statistical significance was defined as a two-tailed p-value of less than 0.05.

### Result

#### Study population characteristics

Of 49,762 patients with T2DM who initiated dialysis for evaluation, the study population was divided into two distinct groups: the SGLT-2i users (*n* = 781, 1.57%) and the non-user (*n* = 48,981, 98.43%), based on the utilization of SGLT-2is within the first three months after the dialysis. The median follow-up period for the entire cohort was 2.0 (IQR, 0.3–3.9) years. Before PSM, the major race was Asian in both groups. SGLT-2i users had a lower percentage of White (17.4% vs. 32.5%; SD = 0.347) and Black or African American (11.7% vs. 18.2%; SD = 0.181); however, a higher percentage of Asian (64.7% vs. 33.6%; SD = 0.642) and not Hispanic or Latino (91.9% vs. 72.2%; SD = 0.529) compared to non-users. Additionally, SGLT-2i users had a higher percentage of male patients (65.6% vs. 56.6%; SD = 0.179). SGLT-2i users had lower rates of nicotine dependence and CKD but higher rates of cardiovascular comorbidities and neoplasms. They were more frequently prescribed glucose-lowering, antiplatelet, and antihypertensive drugs. Additionally, obesity, poor sugar control, and better kidney function were observed among SGLT-2i users. After PSM, both groups were well balanced in each covariate, with all standardized differences less than 0.1 (Table 1). The number of patients excluded due to the absence of any follow-up after the index date was 10 out of 771 (1.3%) in the SGLT-2i users and 10 out of 771 (1.3%) in the non-users (detailed in Table S1). The reasons for initiating dialysis are detailed in Table S2, with the advanced CKD accounting for 23.9% and 24.3% of cases, respectively. Specifically, AKI primarily stems from heart failure (37.0%) and sepsis (23.2%).

**Table 1:** Baseline characteristics between patients using SGLT-2is and non-users before and after propensity score matching

	Before matching			After matching		
	SGLT-2i users (*n* = 781)	Non-users (*n* = 48,981)	Sth diff	SGLT-2i users (*n* = 771)	Non-users (*n* = 771)	Sth diff
Age, mean ± SD	63.3 ± 12.3	63 ± 12.8	0.027	63.3 ± 12.3	63.1 ± 12.9	0.017
Sex, n (%)						
Male	512 (65.6%)	27,689 (56.6%)	0.179	502 (65.1%)	507 (65.8%)	0.014
Female	269 (34.4%)	21,292 (43.4%)	0.179	269 (34.9%)	264 (34.2%)	0.014
Ethnicity, n (%)						
Not Hispanic or Latino	718 (91.9%)	35,349 (72.2%)	0.529	708 (91.8%)	723 (93.8%)	0.075
Hispanic or Latino	31 (4.0%)	5,030 (10.3%)	0.245	31 (4.0%)	27 (3.5%)	0.027
Unknown ethnicity	32 (4.1%)	8,602 (17.6%)	0.441	32 (4.2%)	21 (2.7%)	0.078
Race, n (%)						
American Indian or Alaska	10 (1.3%)	183 (0.4%)	0.101	10 (1.3%)	10 (1.3%)	< 0.001
Asian	505 (64.7%)	16,465 (33.6%)	0.642	495 (64.2%)	495 (64.2%)	< 0.001
Black or African American	91 (11.7%)	8,905 (18.2%)	0.181	91 (11.8%)	83 (10.8%)	0.033
Native Hawaiian or other Pacific Islander	10 (1.3%)	483 (1.0%)	0.029	10 (1.3%)	10 (1.3%)	< 0.001
White	136 (17.4%)	15,920 (32.5%)	0.347	136 (17.6%)	146 (18.9%)	0.034
Unknown race	39 (5.0%)	7,208 (14.7%)	0.318	39 (5.1%)	37 (4.8%)	0.002
Daily behaviors and habits, n (%)						
Nicotine dependence	81 (10.3%)	6,602 (13.5%)	0.006	81 (10.5%)	80 (10.4%)	< 0.001
Tobacco use	10 (1.3%)	518 (1.1%)	0.022	10 (1.3%)	10 (1.3%)	< 0.001
Alcohol-related disorders	20 (2.6%)	861 (1.8%)	0.057	19 (2.5%)	21 (2.7%)	0.016
Comorbidities, n (%)						
Hypertensive diseases	563 (72.1%)	35,783 (73.1%)	0.008	560 (72.6%)	554 (71.9%)	0.017
Ischemic heart diseases	277 (35.5%)	13,628 (27.8%)	0.171	274 (35.5%)	268 (34.8%)	0.016
Peripheral vascular diseases	131 (16.8%)	7,613 (15.6%)	0.037	130 (16.9%)	119 (15.4%)	0.039
Cerebrovascular diseases	158 (20.2%)	6,206 (12.7%)	0.209	158 (20.5%)	163 (21.1%)	0.016
COPD	84 (10.8%)	3,996 (8.2%)	0.092	83 (10.8%)	77 (10.0%)	0.026
Asthma	40 (5.1%)	2,302 (4.7%)	0.022	40 (5.2%)	35 (4.5%)	0.030
Chronic kidney disease	327 (41.9%)	33,173 (67.8%)	0.531	325 (42.2%)	304 (39.4%)	0.055
Dementia	13 (1.7%)	1,052 (2.1%)	0.031	13 (1.7%)	12(1.6%)	0.002
Sleep disorders	138 (17.7%)	6,954 (14.2%)	0.099	137 (17.8%)	128 (16.6%)	0.031
Depressive episodes	55 (7.0%)	3,782 (7.7%)	0.024	54 (7.0%)	45 (5.8%)	0.048
Anxiety disorders	55 (7.0%)	3,406(7.0%)	0.005	54 (7.0%)	47 (6.1%)	0.037
Neoplasms	229 (29.3%)	10,617 (21.7%)	0.182	229 (29.7%)	231 (30.0%)	0.006
Medications, n (%)						
Sulfonylureas	135 (17.3%)	4,361 (8.9%)	0.254	134 (17.4%)	139 (17.5%)	0.007
DPP4i	140 (17.9%)	4,172 (8.5%)	0.285	139 (18.0%)	154 (19.9%)	0.049
GLP-1 analogues	46 (5.9%)	907 (1.9%)	0.216	43 (5.6%)	27 (3.5%)	0.098
Thiazolidinedione	60 (7.7%)	1,049 (2.1%)	0.261	57 (7.4%)	63 (8.2%)	0.030
Insulin	419 (53.6%)	21,168 (43.2%)	0.219	416 (54.0%)	417 (54.1%)	0.003
Aspirin	259 (33.2%)	12,328 (25.2%)	0.183	257 (33.3%)	247 (32.0%)	0.028
Clopidogrel	139 (17.8%)	4,718 (9.6%)	0.243	137 (17.8%)	135 (17.5%)	0.007
Statins	393 (50.3%)	18,094 (40.0%)	0.281	390 (50.6%)	391 (50.7%)	0.003
Allopurinol	35 (4.5%)	2,383 (4.9%)	0.016	35 (4.5%)	35 (4.5%)	< 0.001
Febuxostat	28 (3.6%)	676 (1.4%)	0.144	28 (3.6%)	19 (2.5%)	0.068
Alpha-blocker	156 (20.0%)	6,326 (13.0%)	0.196	156 (20.2%)	153 (19.8%)	0.010
Beta-blocker	339 (43.4%)	19,940 (40.7%)	0.062	337 (43.7%)	345 (44.7%)	0.021
CCB	356 (45.6%)	18,075 (36.9%)	0.185	355 (46.0%)	344 (44.6%)	0.029
ACEI/ARB	334 (42.7%)	13,160 (26.9%)	0.373	341 (44.2%)	349 (45.3%)	0.021
Clinical measures						
BMI	28.1 ± 7.13	29 ± 7.22	0.124	28.1 ± 7.11	28.1 ± 6.59	0.007
≥ 30 kg/m2	247 (31.6%)	18,210 (37.2%)	0.116	244 (31.6%)	256 (33.2%)	0.034
25–30 kg/m2	265 (33.9%)	16,159 (33.0%)	0.018	261 (33.9%)	260 (33.8%)	0.002
<25 kg/m2	269 (34.4%)	14,613 (29.8%)	0.099	266 (34.5%)	254 (33.0%)	0.032
SBP, mm [Hg]	130 ± 22.2	135 ± 25.3	0.211	130 ± 22.2	129 ± 23.7	0.013
Laboratory results						
eGFR	68.8 ± 33.5	47.3 ± 40	0.585	68.8 ± 33.5	69.1 ± 36.9	0.008
≥ 60 mL/min/1.73m2	271 (34.8%)	12,530 (25.6%)	0.202	269 (34.9%)	276 (35.7%)	0.018
45–59 mL/min/1.73m2	196 (25.1%)	7212 (14.7%)	0.260	193 (25.0%)	193 (25.0%)	< 0.001
30–44 mL/min/1.73m2	151 (19.4%)	6834 (14.0%)	0.145	149 (19.4%)	140 (18.1%)	0.031
15–29 mL/min/1.73m2	102 (13.0%)	9343 (19.4%)	0.165	101 (13.1%)	104 (13.5%)	0.011
< 15 mL/min/1.73 m	61 (7.8%)	13,062 (26.7%)	0.518	59 (7.7%)	59 (7.7%)	< 0.001
Proteinuria, mg/dL	4.41 ± 26.2	7.64 ± 43.6	0.090	4.42 ± 26.3	20.6 ± 44.9	0.016
UPCR						
< 30 mg/g	231 (29.6%)	24,086 (49.2%)	0.406	229 (29.7%)	225 (29.2%)	0.011
30–299 mg/g	223 (28.6%)	9,090 (18.6%)	0.239	221 (28.7%)	227 (29.4%)	0.017
≥ 300 mg/g	327 (41.9%)	15,805 (32.3%)	0.202	321 (41.6%)	319 (41.4%)	0.005
Total cholesterol, mg/dL	159 ± 55.6	158 ± 49.5	0.028	160 ± 55.7	157 ± 45.7	0.046
HbA1c	8.01 ± 2.02	7.22 ± 1.87	0.405	8.01 ± 2.03	7.9 ± 2.1	0.049
≥ 7.5%	362 (46.4%)	15,245 (31.1%)	0.317	356 (46.2%)	353 (45.8%)	0.008
6.5–7.5%	223 (28.6%)	15,602 (31.9%)	0.072	221 (28.6%)	233 (30.3%)	0.034
<6.5%	196 (25.1%)	18,135 (37.0%)	0.261	194 (25.2%)	185 (24.0%)	0.027
ALT, units/L	32.8 ± 45.8	35.4 ± 146	0.025	32.7 ± 45.8	38.7 ± 84.2	0.089
Potassium, mEq/L	4.13 ± 0.53	4.22 ± 0.62	0.163	4.13 ± 0.53	4.15 ± 0.56	0.042
BNP, pg/mL	1,057 ± 2,355	1,327 ± 3,727	0.086	1,060 ± 2,360	1,183 ± 3,401	0.042
Peritoneal dialysis, n (%)	17 (2.2%)	961 (2.0%)	0.016	17 (2.2%)	19 (2.5%)	0.017
Prior hospitalization, n (%)	291 (37.3%)	9,849 (20.1%)	0.172	291 (37.7%)	298 (38.7%)	0.017

*ACEI* angiotensin converting enzyme inhibitors, *ARB* angiotensin receptor blockers, *ALT* alanine aminotransferase, *BMI* body mass index, *BNP* B-type natriuretic peptide, *CCB* calcium channel blocker, *COPD* chronic obstructive pulmonary disease, *DPP4i* dipeptidyl peptidase-4 inhibitor, *eGFR* estimated Glomerular filtration rate, *GLP-1* glucagon-like peptide 1, *HbA1c* glycated hemoglobin, *SBP* systolic blood pressure, *SD* standard deviation, *Std diff* Standardized difference, *SGLT-2i* sodium–glucose cotransporter 2 inhibitor, *UPCR* urine Protein and Creatinine Ratio

### The impact of SGLT-2is on all-cause mortality, MACEs and other outcomes

**Fig. 2:**
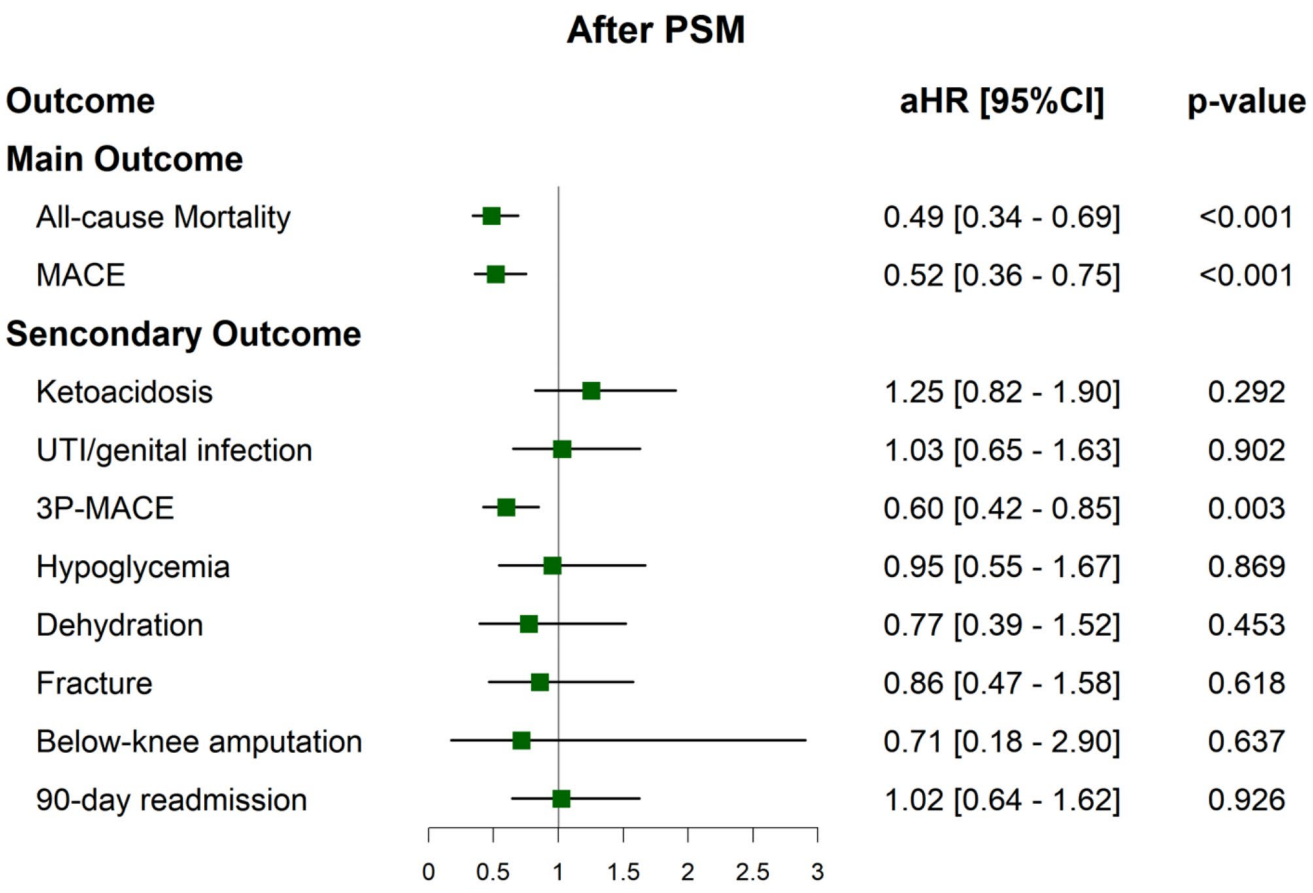
Comparison of the pre-specified outcomes of patients treated with SGLT-2is versus those non-users after prosperity score matching. The forest plots illustrated the adjusted HRs of all-cause mortality, MACE, and other secondary outcomes for SGLT-2i users versus non-users after propensity score matching. The plots present both the adjusted HRs and their 95% confidence intervals (CIs), represented as error bars. The vertical line denotes an aHR of 1.00, with lower limits of the 95% CIs exceeding 1.00 indicating a statistically significant increased risk. *aHR* adjust hazard ratio, *3p-MACE* 3-piont major adverse cardiac event, *MACE* major adverse cardiac event, *PS* propensity score, *UTI* urinary tract infection

**Fig. 3:**
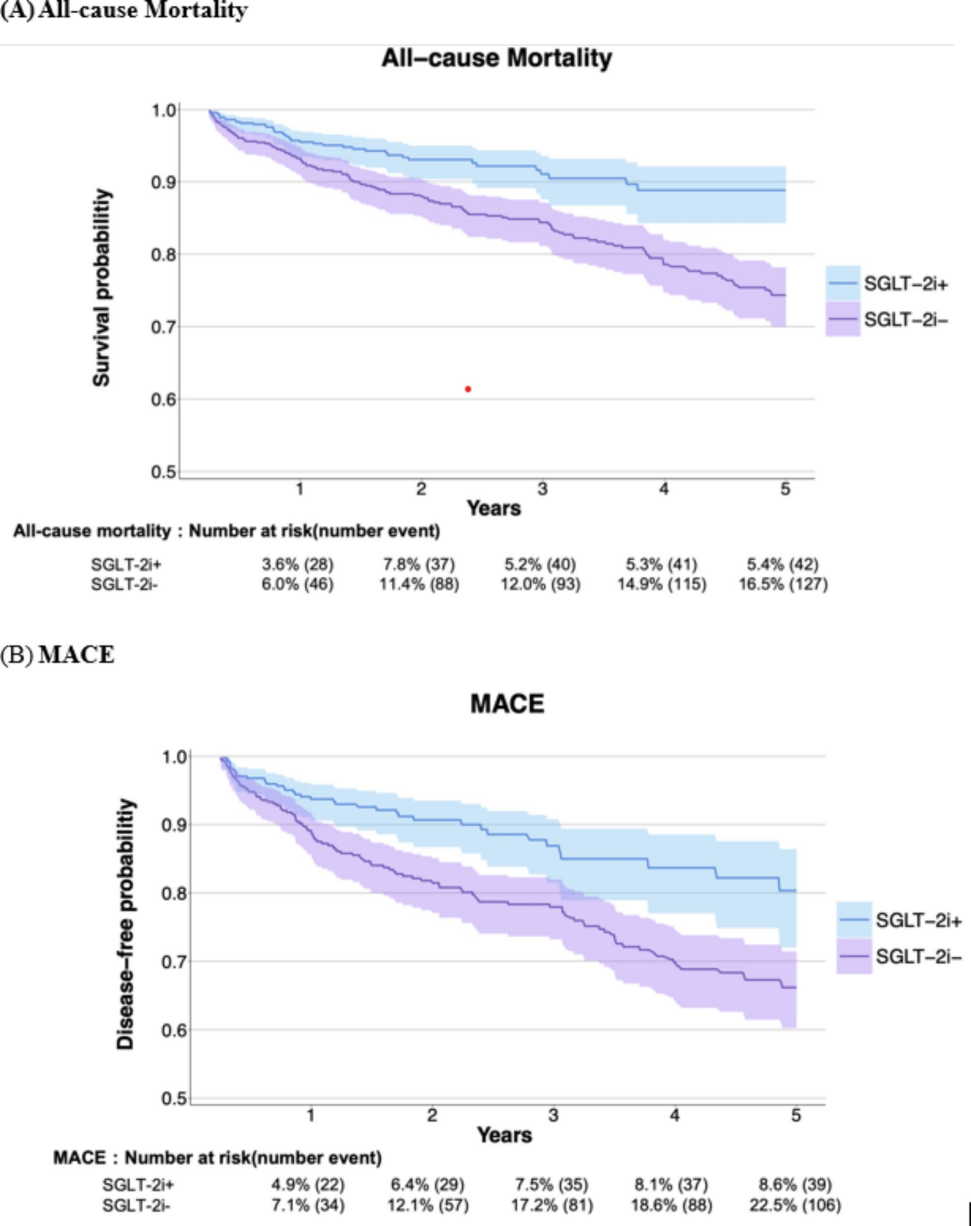
Kaplan-Meier curves of the pre-specified long-term outcome. The blue curve represents individuals who are SGLT-2i users, while the purple curve represents those who are SGLT-2i non-users. Shaded areas indicate 95% CIs. (A) All-cause mortality (log-rank *P* < 0. 001). (B) MACE (log-rank *P* < 0. 001). *MACE* major adverse cardiac event, *SGLT-2is* sodium–glucose cotransporter 2 inhibitors

During follow-up period, 42 (5.4%) patients in the SGLT-2i users and 127 (16.5%) patients in the non-users died, while 39 (8.6%) patients in the SGLT-2i users and 106 (22.5%) patients in the non-users experienced MACE. We found a significantly lower hazard of all-cause mortality (adjusted hazard ratio (aHR) = 0.49; 95% CI = 0.34–0.69, *p* < 0.001) and MACE (aHR = 0.52; 95% CI = 0.36–0.75, *p* < 0.001) in SGLT-2i users compared with non-users (Figs. 2 and 3, Figure S1, and Table S3). The risk difference of mortality was − 11% between SGLT-2i users and non-users (95% CI=-0.14- -0.08, *p* < 0.001) (Table S4-5). Analysis of the individual elements of MACE revealed that cardiovascular death/mortality contributed the most to the overall MACE outcome (aHR = 0.47; 95% CI = 0.33–0.68, *p* < 0.001) (Table S6). The *E*-value for all-cause mortality was 3.54, larger than the upper limit of the confidence interval at 2.26. For MACE, the *E*-value was 3.28, larger than the upper limit of the confidence interval at 2.01 (Table S3). No significant differences were observed in the outcomes of ketoacidosis, UTI or genital infection, hypoglycemia, dehydration, bone fracture, below-knee amputation, and 90-day readmission (Fig. 2 and Table S7). SGLT-2i users had a significantly higher likelihood of being free from dialysis compared to non-users in 90 days after the index date, with 95.1% of SGLT-2i users achieving dialysis-free status compared to 89.5% of non-users (aHR = 0.49; 95% CI = 0.33–0.73, *p* < 0.001). Additionally, only a very limited number of patients in the SGLT-2i group (less than 10) underwent kidney transplantation during the study period, while no patients in the non-SGLT-2i group received a kidney transplant (Table S8).

### Negative outcome, positive and negative exposure controls

Our study revealed that there were no significant associations between SGLT-2is use and the incidence of skin cancer, herniated disc, hemorrhoids, COPD, upper respiratory infections, and GERD. The results indicated that the use of SGLT-2is was not significantly associated with a heightened risk of any of these outcomes, which aligns with prior knowledge and expectations [32, 33] (Figure S2).

Based on literature suggesting that use of ARB is linked to a reduced risk of all-cause mortality and MACE. Our findings supported these reports, showing that ARB users had a significantly lower hazard of all-cause mortality and MACE compared to non-users. Results were consistent when H2 blockers and antidepressants (SSRIs) were introduced as negative exposure controls. (Figure S2)

### Subgroup and sensitivity analysis

**Fig. 4:**
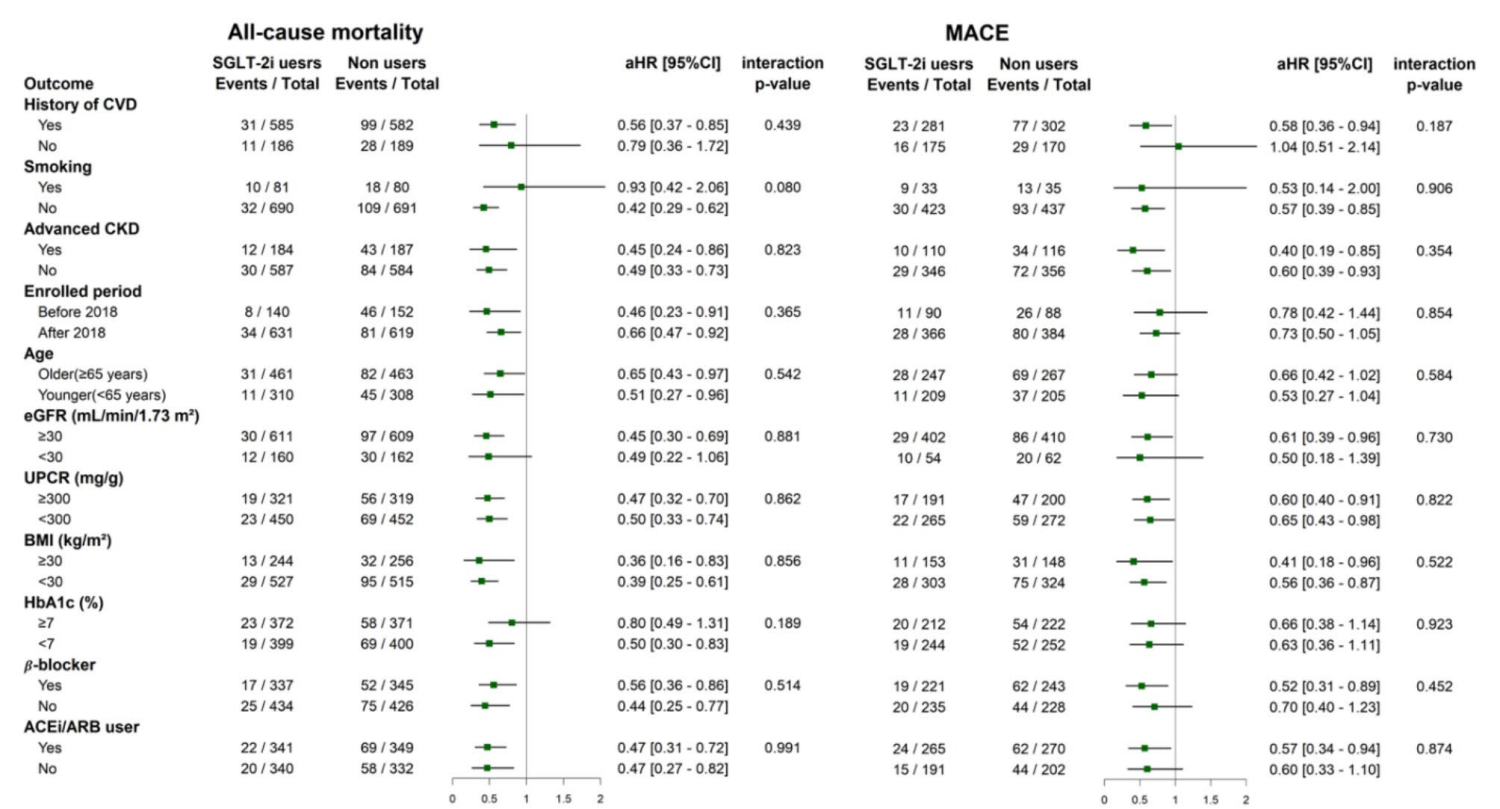
Subgroup analysis. The forest plots illustrated the adjusted HRs of all-cause mortality and MACE for SGLT-2is users versus non-users across various subgroups. The plots present both the adjusted HRs and their 95% confidence intervals (CIs), represented as error bars. The vertical line denotes an aHR of 1.00, with lower limits of the 95% CIs exceeding 1.00 indicating a statistically significant increased risk. Advanced CKD defined as baseline kidney function less than eGFR 15 ml/min/1.73^2^. *ACEi* angiotensin converting enzyme inhibitors, *ARB* angiotensin receptor blockers, *BMI* body mass index, *CI* confidence interval, *CKD* chronic kidney disease, *CVD* cardiovascular diseases, *eGFR* estimated, glomerular filtration rate, *HbA1c* Glycated Hemoglobin, *aHR* adjusted hazard ratio, *MACE* major adverse cardiac event, *SGLT-2i* sodium–glucose cotransporter 2 inhibitor

We conducted subgroup analyses to assess the influence of various factors on study outcomes. These analyses included baseline characteristics (eGFR, UPCR, and HbA1c levels, BMI, CVD, advanced CKD, and current use of beta blockers or ACEI/ARB), demographic factors (smoking status and age) and participants enrolled before or after 2018 (Fig. 4). These results showed that SGLT-2is were associated with reduced hazard of mortality and MACE across various subgroups. However, none of the interaction p-values were greater than 0.05, indicating that these associations were consistent across all subgroups without significant variation.

For the sensitivity analysis, we examined the effects of varying follow-up durations, as well as different types of SGLT-2 inhibitors used (Table S9 and Figure S3). Landmark analysis further confirmed that setting the different timeframe of selection period within 14, 30, 60 days produced consistent results (Table S10). We also employed various Cox proportional hazards regression models with different covariates, all of which consistently aligned with our primary approach (Table S11-12). The extended analysis of SGLT-2is discontinuation timing, comparing continued use versus discontinuation within 3 months after the index date, also showed consistent outcomes (Table S13).

## Discussion

Our analysis suggested that among T2DM patients at dialysis initiation, the new users of SGLT-2i could be linked with a reduction in the risk of all-cause mortality, and MACE over a median follow-up period of 2.0 years. Our study did not observe significant differences in the incidence of ketoacidosis, hypoglycemia, below-knee amputations, bone fractures, UTI or genital infection, dehydration, or 90-day readmission when compared to patients not using SGLT-2is. Notably, SGLT-2i users had a higher likelihood of achieving dialysis-free status at 90 days.

Several current studies have shown that SGLT-2is improve cardiovascular outcomes in patients with T2DM [8, 10, 41, 42]. Clinical trials have established that SGLT-2is reduce the risk of renal disease progression and death from renal causes in patients with T2DM, as well as in those with CKD, regardless of their diabetes status [14, 41, 43]. SGLT-2is improve glucose control primarily by promoting glucosuria, which leads to increased insulin sensitivity and enhanced beta-cell function [44]. Beyond glycemic control, these inhibitors exert pleiotropic effects that extend to cardiovascular benefits [44–46]. The combined effect of glucose-induced osmotic diuresis and natriuresis contributes to a decrease in cardiac preload while the reduction in arterial stiffness and systemic blood pressure aids in diminishing afterload [47]. However, the exact mechanisms underlying the persistent cardiovascular benefits of SGLT-2is in patients on dialysis or with severely impaired kidney function are multifaceted and not fully understood [48].

Potential mechanisms include the inhibition of the cardiac sodium-hydrogen exchanger, which contributes to the amelioration of cardiac hypertrophy, fibrosis, and injury [49]. This concept of SGLT-2 transporter-independent cardiac benefits is supported by a bioinformatic study that used in silico modeling of RNA sequence datasets from cardiac tissues of diabetic rats treated with empagliflozin [50]. Enhancing ketogenic nutrient deprivation signaling through the upregulation of the SIRT1/PGC-1α/FGF21 pathway leads to alleviation of oxidative stress/inflammation, augmentation of autophagic flux, and increased erythropoiesis, which may contribute to improved cardiovascular outcomes and overall cellular health [51]. SGLT-2is have demonstrated beneficial of preventing adverse cardiac remodeling. In a randomized trial, change in LV mass index was shown in people with T2DM who treated with empagliflozin [52]. Collectively, these potential mechanisms are independent of proximal tubular SGLT-2 and contribute to the improvement of cardiovascular events in patients with minimal diuresis.

Our study demonstrates that T2DM patients initiating dialysis who were treated with SGLT-2is had a higher likelihood of achieving dialysis-free status at 90 days. In the post-hoc analysis from EMPA-KIDNEY trial, 245 participants were with eGFR less than 20 mL/min/1.73 m², 27% relative risk reduction in the incidence of the progression of kidney disease was consistent with the effect size in the overall population [15]. These exploratory subgroup data support the hypothesis that SGLT-2is may exert beneficial effects in patients at advanced stages of CKD. The kidney-protective effects of SGLT-2is are believed to operate through multiple mechanisms [53]. They reduce intraglomerular pressure by restoring tubuloglomerular feedback and may also exert anti-inflammatory effects and enhance mitochondrial function, collectively contributing to reduced fibrosis and oxidative stress in the kidney [45, 54].

Safety concerns regarding the use of SGLT-2is in patients with T2DM on dialysis are important. Our study suggested that SGLT-2is were not associated with ketoacidosis, hypoglycemia, below-knee amputations, bone fractures, UTI or genital infection, or dehydration. A retrospective study on seven patients with diabetes undergoing intermittent hemodialysis (iHD) over 12 months found SGLT-2is treatment to be safe, with no reported cases of euglycemic ketoacidosis, bone fractures, or amputations [55]. Further research by Barreto et al. has provided insights into the pharmacokinetics of dapagliflozin in individuals with kidney failure undergoing hemodialysis or peritoneal dialysis [56]. The findings suggest that while dapagliflozin is not dialyzable, significant drug accumulation was not observed, and no serious adverse events were reported, though the follow-up duration was short. Specifically, the DAPA-CKD trial offered insights into the use of SGLT-2is in patients with kidney failure [43]. In an exploratory analysis of 167 participants who progressed to chronic dialysis, the rates of serious adverse events were comparable between those treated with dapagliflozin and those given a placebo. However, this analysis did not specifically address the cardiovascular benefits and kidney protection associated with SGLT-2 inhibitors [19]. Consequently, further randomized clinical trials are necessary to validate these findings and explore these potential benefits in greater depth.

The consistent results across prespecified subgroups support the strength of our findings. Our study highlights the potential association between new SGLT-2i users and cardiovascular outcomes in patients with T2DM who initiated dialysis, marking an initial step in understanding this relationship. The new-users design employed in our study ensures the data’s relevance to patients starting SGLT-2is, thereby enhancing the validity of our findings. Utilizing real-world data, as demonstrated in our research, provides unique advantages, offering a broad perspective on patient information that is crucial for informing future treatment approaches and research efforts.

Our studies did have some limitations. First, the predominance of Asian participants in our study may limit the generalizability of our findings. Second, the inherent nature of retrospective designs and the potential for misclassification bias and residual confounding cannot be completely eliminated. To evaluate the influence of potential unmeasured confounding, we conducted an *E*-value analysis, as well as PSM and variable models of multivariate Cox proportional analysis. The findings suggest that it is unlikely for an unmeasured confounder to exert a more significant effect on the primary outcome than the use of SGLT-2is. Third, the limited number of patients initiating dialysis who used SGLT-2is within our study cohort could affect the robustness of our results. The small sample size of our cohort inherently limits the statistical power to detect heterogeneity, increasing the risk of type 2 error. Additionally, the process of selecting an appropriate control group from a large cohort may lead to challenges in finding suitable matches. This can result in increased sample imbalance and potential bias. Fourth, the shorter follow-up period limits our ability to assess long-term outcomes and sustained effects of SGLT-2is, reflecting real-world practices where these drugs are selectively prescribed. Hazard ratios alone may not fully capture clinical significance [57], so we also present absolute risks and risk differences to provide a clearer view of the potential clinical relevance. Fifth, limitations related to the dataset include its aggregated nature, which restricts the ability to trace reasons for discontinuation of prescriptions and limits the application of advanced statistical methods. Due to the constraints of the TriNetX platform, we were unable to perform either multiple rounds of PSM or competing risk analysis to enhance model precision and minimize bias. Additionally, the dataset does not provide the precise dates of dialysis initiation and/or discontinuation, or renal transplant, thereby precluding the use of time-varying models. Sixth, our study did not specifically consider the dosage of SGLT-2is. While the effects of SGLT-2is are generally not considered dose-dependent, future research may further explore this aspect to confirm consistency across different dosages. Finally, it is important to note that our study was centered on patients with T2DM at the new onset of dialysis. Our landmark analysis ensured consistent results, mitigating the possibility of guarantee-time bias or immortal time bias [58]. As a result, our findings may not extend to patients on long-term dialysis. In light of these limitations, cautious interpretation of our findings is warranted, and further research is needed to validate and expand upon our observations.

## Conclusions

Our real-world study suggested that new SGLT-2i users in T2DM patients at the onset of dialysis were associated with a reduced long-term risk of all-cause mortality and MACE over a median follow-up of 2.0 years. Additionally, they would have a higher likelihood of achieving dialysis-free status at 90 days without an increased risk of serious adverse events such as ketoacidosis, hypoglycemia, or infections. Further randomized clinical trials are essential to fully validate these findings and explore the cardiovascular and kidney protective effects in this population.

Data availability The datasets used and/or analyzed during the current study are available from the corresponding author on reasonable request.

## Electronic supplementary material

Supplementary Material 1

Supplementary Material 2

## Data Availability

The datasets used and/or analyzed during the current study are available from the corresponding author on reasonable request.
