# Supplementary material for: Exploring the mortality and cardiovascular outcomes with SGLT-2 inhibitors in patients with T2DM at dialysis commencement: a health global federated network analysis
Source: Cardiovasc Diabetol. 2024 Sep 3;23:327. doi: 10.1186/s12933-024-02424-7 (PMC11373240; doi:10.1186/s12933-024-02424-7)
Supplement: Supplementary file 1 — Supplementary Material 1 [file 12933_2024_2424_MOESM1_ESM.docx]

Supplemental Content to “Exploring the mortality and cardiovascular outcomes with SGLT-2 Inhibitors in Patients with T2DM at Dialysis Commencement: A Health Global Federated Network Analysis”

Graphic Abstract

Study Design

Supplementary Method.

Definition of Variables

Definition of Outcomes

Table S1. Numbers and Demographics of Individuals Excluded Because of a Lack of Any Follow-Up

Table S2. Presumptive causes of dialysis

Table S3. Incidence rate and E-values of primary outcomes among SGLT-2i users compared to non-users after prosperity score matching

Table S4. Risk of mortality in patients with T2DM at dialysis commencement: comparison between SGLT-2i users and non-users after propensity score matching

Table S5. Risk of MACE in patients with T2DM at dialysis commencement: comparison between SGLT-2i users and non-users after propensity score matching

Table S6. Incidence Rate of MACE Subcomponents Among SGLT-2i Users Compared to Non-Users After Propensity Score Matching

Table S7. Incidence rate of among SGLT-2i users compared to non-users after prosperity score matching

Table S8. Landmark analysis of SGLT-2 Inhibitors in T2DM Patients after Dialysis Initiation: Focus on Kidney Outcomes

Table S9. Incidence of pre-specified outcomes with different follow-up time between SGLT-2i Users and Non-users

Table S10. Landmark analysis for primary outcomes across varied durations of SGLT-2is use

Table S11. Sensitivity Analysis for All-Cause Mortality Between SGLT-2 is Users and Non-users

Table S12. Sensitivity Analysis for MACE Between SGLT-2is Users and Non-users

Table S13. Sensitivity Analysis of SGLT-2 Inhibitors usage Status After Index Date

Figure S1. Comparison of the pre-specified outcomes of patients treated with SGLT-2is versus those non-users. before prosperity score matching

Figure S2. Negative outcome, positive and negative exposure controls

Figure S3. Sensitivity study on various types and dosages of SGLT-2is

STROBE Statement—checklist of items that should be included in reports of observational studies

**Graphic Abstract**


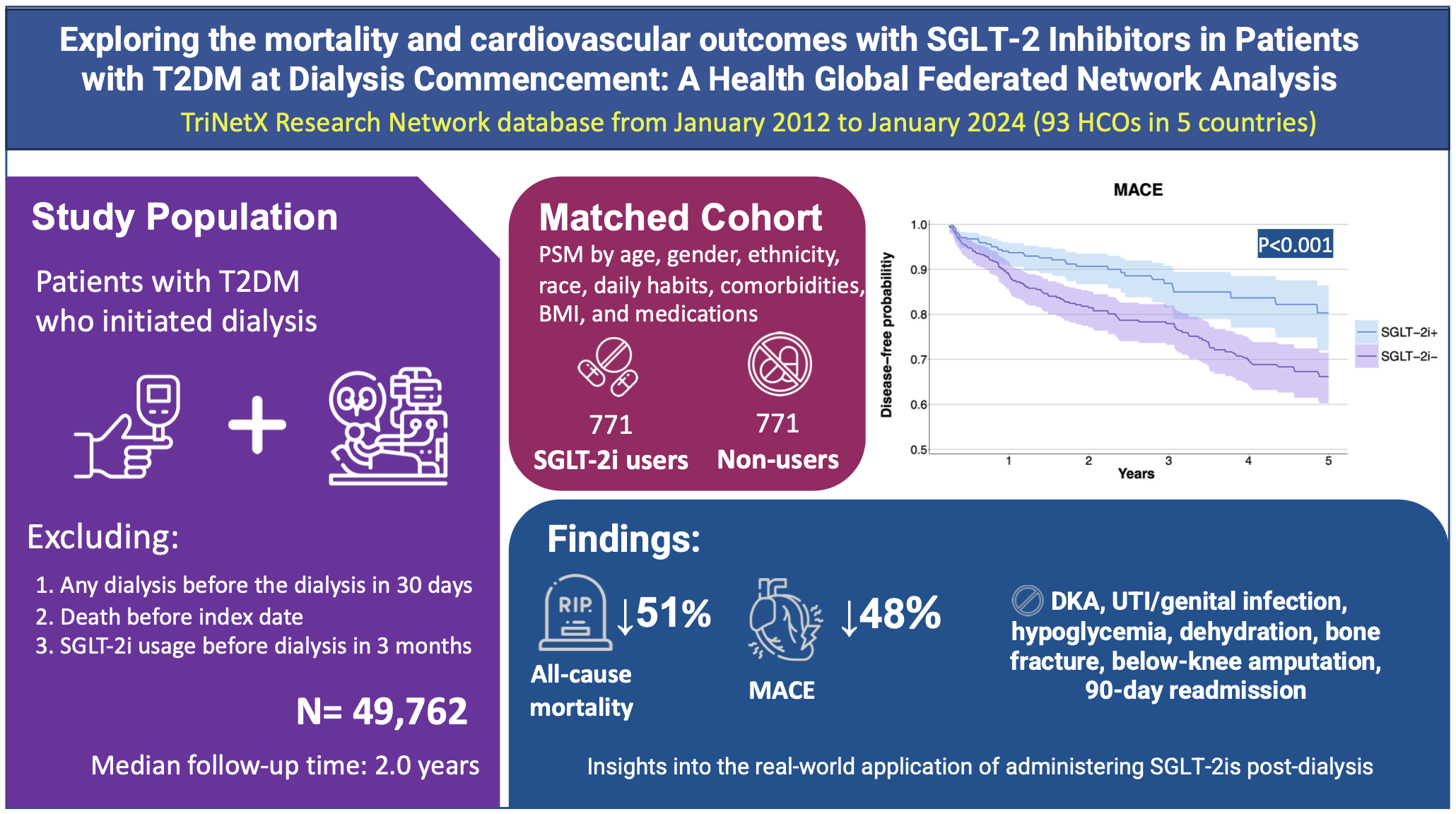


**Study Design**


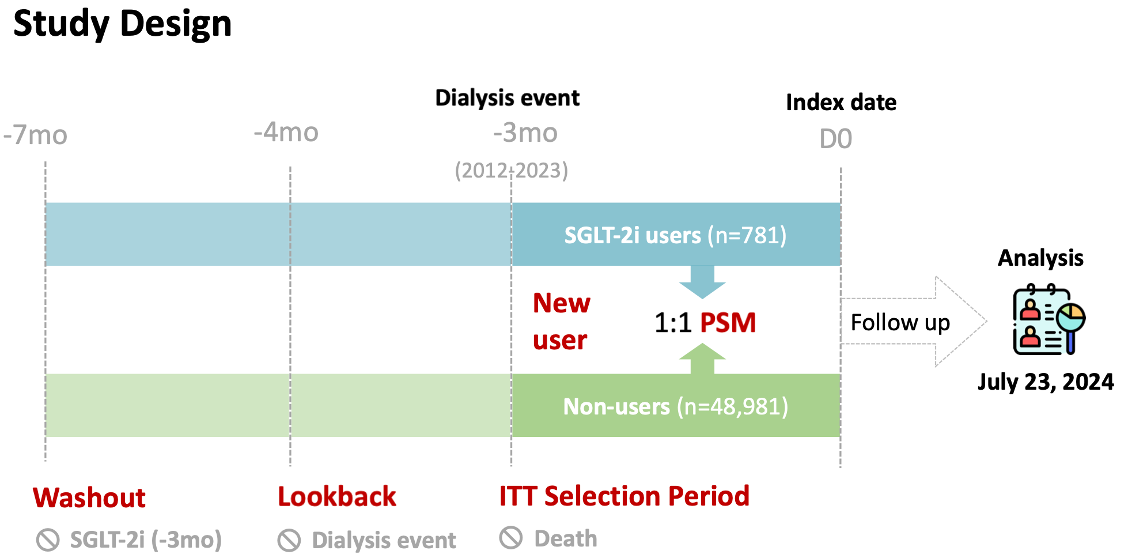


**Supplementary Method.**

**Definition of Variables**

| **Clinical measures** | | | |
| --- | --- | --- | --- |
| Blood Pressure, Systolic | TNX Curated 9085 | BMI | TNX Curated 9083 |
| **Laboratory results** | | | |
| Glomerular filtration rate/1.73 sq M.predicted [Volume Rate/Area] in Serum, Plasma or Blood by Creatinine-based formula (MDRD) | TNX Curated 8001 | Hemoglobin [Mass/volume] in Blood | LOINC 718-7 |
| Hemoglobin A1c/Hemoglobin.total in Blood | TNX Curated 9037 | Albumin [Mass/volume] in Serum, Plasma or Blood | TNX Curated 9045 |
| Cholesterol [Mass/volume] in Serum or Plasma | TNX Curated 9000 | Potassium [Moles/volume] in Serum, Plasma or Blood | TNX Curated 9028 |
| Natriuretic peptide B [Mass/volume] in Serum, Plasma or Blood | TNX Curated 9003 | Alanine aminotransferase [Enzymatic activity/volume] in Serum, Plasma or Blood | TNX Curated 9044 |
| Protein [Presence] in Urine | TNX Curated  LG35161-5 | Albumin/Creatinine [Mass ratio] in Urine | TNX Curated  LG34557-5 |
| **Daily behaviors and habits** | | | |
| Nicotine dependence | ICD-10-CM F17 | Tobacco use | ICD-10-CM Z72.0 |
| Alcohol related disorders | ICD-10-CM F10 |  |  |
| **Comorbidities** | | | |
| Ischemic heart diseases | ICD-10-CM I20-I25 | Peripheral vascular diseases | ICD-10-CM I70-I79 |
| Cerebrovascular diseases | ICD-10-CM I60-I69 | Chronic obstructive pulmonary disease | ICD-10-CM J44 |
| Hypertensive diseases | ICD-10-CM  I10-I1A | Asthma | ICD-10-CM J45 |
| Neoplasms | ICD-10-CM C00-D49 | Chronic kidney diseases | ICD-10-CM N18 |
| Dementia | ICD-10-CM F03 | Sleep disorders | ICD-10-CM G47 |
| Depressive episode | ICD-10-CM  F32 | Anxiety disorders | ICD-10-CM F41 |
| **Drugs** | | | |
| Sulfonylureas | ATC A10BB | Dipeptidyl peptidase 4 (dpp-4) inhibitors | ATC A10BH |
| Glucagon-like peptide-1 (glp-1) analogues | ATC A10BJ | ACEI/ARB | ATC C09 |
| Insulins | ATC A10A | Aspirin | RxNorm 1191 |
| Clopidogrel | RxNorm 32968 | Allopurinol | RxNorm 519 |
| Febuxostat | RxNorm 73689 | Beta blockers | VA CV100 |
| Alpha blockers | VA CV150 | Calcium channel blockers | VA CV200 |
| Thiazolidinediones | ATC A10BG | Statins | ATC C10AA |

**Definition of Outcomes**

| Mortality | | | | |
| --- | --- | --- | --- | --- |
|  | **Outcome definition** | | | |
|  | | Diagnosis | UMLS:ICD10CM:R99 | Ill-defined and unknown cause of mortality |
|  | | Demographics | Deceased | Deceased |
|  | | Diagnosis | UMLS:ICD10CM:R99-R99 | Ill-defined and unknown cause of mortality (R99) |
|  | | Diagnosis | UMLS:ICD10CM:R69 | Illness, unspecified |

| MACE | | | | |
| --- | --- | --- | --- | --- |
|  | **Outcome definition** | | | |
|  | | Demographics | Deceased | Deceased |
|  | | Diagnosis | UMLS:ICD10CM:I46 | Cardiac arrest |
|  | | Diagnosis | UMLS:ICD10CM:I21 | Acute myocardial infarction |
|  | | Diagnosis | UMLS:ICD10CM:I63 | Cerebral infarction |
|  | | Diagnosis | UMLS:ICD10CM:R99 | Ill-defined and unknown cause of mortality |
|  | | Diagnosis | UMLS:ICD10CM:R99-R99 | Ill-defined and unknown cause of mortality (R99) |
|  | | Diagnosis | UMLS:ICD10CM:R69 | Illness, unspecified |
|  | | Diagnosis | UMLS:ICD10CM:I61 | Nontraumatic intracerebral hemorrhage |
|  | | Diagnosis | UMLS:ICD10CM:I20 | Angina pectoris |

| Ketoacidosis | | | | |
| --- | --- | --- | --- | --- |
|  | **Outcome definition** | | | |
|  | | Diagnosis | UMLS:ICD10CM:E11.1 | Type 2 diabetes mellitus with ketoacidosis |
|  | | Diagnosis | UMLS:ICD10CM:E13.1 | Other specified diabetes mellitus with ketoacidosis |
|  | | Diagnosis | UMLS:ICD10CM:E13.1 | Other specified diabetes mellitus with ketoacidosis |
|  | | Diagnosis | UMLS:ICD10CM:E11.1 | Type 2 diabetes mellitus with ketoacidosis |
|  | | Diagnosis | UMLS:ICD10CM:E08.1 | Diabetes mellitus due to underlying condition with ketoacidosis |
|  | | Diagnosis | UMLS:ICD10CM:E11.65 | Type 2 diabetes mellitus with hyperglycemia |
|  | | Diagnosis | UMLS:ICD10CM:E87.2 | Acidosis |
|  | |  |  |  |

| 3P-MACE | | | | |
| --- | --- | --- | --- | --- |
| Outcome definition | | | |  |
|  | Demographics | Deceased | Deceased | |
|  | Diagnosis | UMLS:ICD10CM:R69 | Illness, unspecified | |
|  | Diagnosis | UMLS:ICD10CM:R99 | Ill-defined and unknown cause of mortality | |
|  | Diagnosis | UMLS:ICD10CM:R99-R99 | Ill-defined and unknown cause of mortality (R99) | |
|  | Diagnosis | UMLS:ICD10CM:I21 | Acute myocardial infarction | |
|  | Diagnosis | UMLS:ICD10CM:I46 | Cardiac arrest | |
|  | Diagnosis | UMLS:ICD10CM:I61 | Nontraumatic intracerebral hemorrhage | |
|  | Diagnosis | UMLS:ICD10CM:I63 | Cerebral infarction | |

| Hypoglycemia | | | | |
| --- | --- | --- | --- | --- |
|  | **Outcome definition** | | | |
|  | | Diagnosis | UMLS:ICD10CM:E16.2 | Hypoglycemia, unspecified |
|  | | Laboratory | TNX:9025 | Glucose [Mass/volume] in Serum, Plasma or Blood (at most 70.00 mg/dL (most recent occurrence)) |
|  | | Laboratory | UMLS:LNC:2345-7 | Glucose [Mass/volume] in Serum or Plasma (at most 70.00 mg/dL (most recent occurrence)) |

| Dehydration | | | | |
| --- | --- | --- | --- | --- |
|  | **Outcome definition** | | | |
|  | | Diagnosis | UMLS:ICD10CM:E86.0 | Dehydration |

| Fracture | | | | |
| --- | --- | --- | --- | --- |
|  | **Outcome definition** | | | |
|  | | Diagnosis | UMLS:ICD10CM:S02 | Fracture of skull and facial bones |
|  | | Diagnosis | UMLS:ICD10CM:S12 | Fracture of cervical vertebra and other parts of neck |
|  | | Diagnosis | UMLS:ICD10CM:S32 | Fracture of lumbar spine and pelvis |
|  | | Diagnosis | UMLS:ICD10CM:S42 | Fracture of shoulder and upper arm |
|  | | Diagnosis | UMLS:ICD10CM:S52 | Fracture of forearm |
|  | | Diagnosis | UMLS:ICD10CM:S62 | Fracture at wrist and hand level |
|  | | Diagnosis | UMLS:ICD10CM:S72 | Fracture of femur |
|  | | Diagnosis | UMLS:ICD10CM:S82 | Fracture of lower leg, including ankle |
|  | | Diagnosis | UMLS:ICD10CM:S92 | Fracture of foot and toe, except ankle |

| Urinary tract infection | | | | |
| --- | --- | --- | --- | --- |
| Outcome definition | | | |  |
|  | Diagnosis | UMLS:ICD10CM:N39.0 | Urinary tract infection, site not specified | |

| Genital infection | | | | |
| --- | --- | --- | --- | --- |
| Outcome definition | | | |  |
|  | Diagnosis | UMLS:ICD10CM:N76 | Other inflammation of vagina and vulva | |
|  | Diagnosis | UMLS:ICD10CM:N77 | Vulvovaginal ulceration and inflammation in diseases classified elsewhere | |
|  | Diagnosis | UMLS:ICD10CM:N49 | Inflammatory disorders of male genital organs, not elsewhere classified | |
|  | Diagnosis | UMLS:ICD9CM:616.1 | Vaginitis and vulvovaginitis | |
|  | Diagnosis | UMLS:ICD10CM:B37.3 | Candidiasis of vulva and vagina | |
|  | Diagnosis | UMLS:ICD10CM:A54.0 | Gonococcal infection of lower genitourinary tract without periurethral or accessory gland abscess | |
|  | Diagnosis | UMLS:ICD10CM:A54.1 | Gonococcal infection of lower genitourinary tract with periurethral and accessory gland abscess | |
|  | Diagnosis | UMLS:ICD10CM:A54.2 | Gonococcal pelviperitonitis and other gonococcal genitourinary infection | |
|  | Diagnosis | UMLS:ICD10CM:N71.0 | Acute inflammatory disease of uterus | |
|  | Diagnosis | UMLS:ICD10CM:N71.1 | Chronic inflammatory disease of uterus | |
|  | Diagnosis | UMLS:ICD10CM:B37.42 | Candidal balanitis | |
|  | Diagnosis | UMLS:ICD10CM:N45 | Orchitis and epididymitis | |
|  | Diagnosis | UMLS:ICD10CM:N48.1 | Balanitis | |

| Below-knee amputation | | | | |
| --- | --- | --- | --- | --- |
|  | **Outcome definition** | | | |
|  | | Diagnosis | UMLS:ICD10CM:Z89.5 | Acquired absence of leg below knee |
|  | | Diagnosis | UMLS:ICD10CM:Z89.41 | Acquired absence of great toe |
|  | | Diagnosis | UMLS:ICD10CM:Z89.42 | Acquired absence of other toe(s) |
|  | | Diagnosis | UMLS:ICD10CM:Z89.43 | Acquired absence of foot |
|  | | Diagnosis | UMLS:ICD10CM:Z89.44 | Acquired absence of ankle |

| Mortality/remain on dialysis | | | | |
| --- | --- | --- | --- | --- |
| Outcome definition | | | |  |
|  | Diagnosis | UMLS:ICD10CM:R99 | Ill-defined and unknown cause of mortality | |
|  | Demographics | Deceased | Deceased | |
|  | Diagnosis | UMLS:ICD10CM:R99-R99 | Ill-defined and unknown cause of mortality (R99) | |
|  | Diagnosis | UMLS:ICD10CM:R69 | Illness, unspecified | |
|  | Procedure | UMLS:CPT:90935 | Hemodialysis procedure with single evaluation by a physician or other qualified health care professional | |
|  | Procedure | UMLS:CPT:90945 | Dialysis procedure other than hemodialysis (eg, peritoneal dialysis, hemofiltration, or other continuous renal replacement therapies), with single evaluation by a physician or other qualified health care professional | |
|  | Procedure | UMLS:CPT:90947 | Dialysis procedure other than hemodialysis (eg, peritoneal dialysis, hemofiltration, or other continuous renal replacement therapies) requiring repeated evaluations by a physician or other qualified health care professional, with or without substantial revision of dialysis prescription | |
|  | Procedure | UMLS:CPT:1006747 | Hemodialysis Access, Intervascular Cannulation for Extracorporeal Circulation, or Shunt Insertion Procedures on Arteries and Veins | |
|  | Procedure | UMLS:CPT:1012740 | Dialysis Services and Procedures | |
|  | Procedure | UMLS:CPT:1012752 | Hemodialysis Procedures | |
|  | Procedure | UMLS:CPT:1029674 | Dialysis Circuit Procedures | |
|  | Procedure | UMLS:SNOMED:302497006 | Hemodialysis | |
|  | Procedure | UMLS:ICD9CM:39.95 | Hemodialysis | |

| 90-day readmission | | | | |  |
| --- | --- | --- | --- | --- | --- |
| Outcome definition | | | |  |  |
|  | Procedure | UMLS:CPT:99223 | Initial hospital care, per day, for the evaluation and management of a patient, which requires these 3 key components: A comprehensive history; A comprehensive examination; and Medical decision making of high complexity. Counseling and/or coordination of care with other physicians, other qualified health care professionals, or agencies are provided consistent with the nature of the problem(s) and the patient's and/or family's needs. Usually, the problem(s) requiring admission are of high severity. Typically, 70 minutes are spent at the bedside and on the patient's hospital floor or unit. | | |
|  | Procedure | UMLS:CPT:99222 | Initial hospital care, per day, for the evaluation and management of a patient, which requires these 3 key components: A comprehensive history; A comprehensive examination; and Medical decision making of moderate complexity. Counseling and/or coordination of care with other physicians, other qualified health care professionals, or agencies are provided consistent with the nature of the problem(s) and the patient's and/or family's needs. Usually, the problem(s) requiring admission are of moderate severity. Typically, 50 minutes are spent at the bedside and on the patient's hospital floor or unit. | | |
|  | Procedure | UMLS:CPT:99221 | Initial hospital care, per day, for the evaluation and management of a patient, which requires these 3 key components: A detailed or comprehensive history; A detailed or comprehensive examination; and Medical decision making that is straightforward or of low complexity. Counseling and/or coordination of care with other physicians, other qualified health care professionals, or agencies are provided consistent with the nature of the problem(s) and the patient's and/or family's needs. Usually, the problem(s) requiring admission are of low severity. Typically, 30 minutes are spent at the bedside and on the patient's hospital floor or unit. | | |
|  | Procedure | UMLS:CPT:99220 | Initial observation care, per day, for the evaluation and management of a patient, which requires these 3 key components: A comprehensive history; A comprehensive examination; and Medical decision making of high complexity. Counseling and/or coordination of care with other physicians, other qualified health care professionals, or agencies are provided consistent with the nature of the problem(s) and the patient's and/or family's needs. Usually, the problem(s) requiring admission to outpatient hospital "observation status" are of high severity. Typically, 70 minutes are spent at the bedside and on the patient's hospital floor or unit. | | |
|  | Procedure | UMLS:CPT:99217 | Observation care discharge day management (This code is to be utilized to report all services provided to a patient on discharge from outpatient hospital "observation status" if the discharge is on other than the initial date of "observation status." To report services to a patient designated as "observation status" or "inpatient status" and discharged on the same date, use the codes for Observation or Inpatient Care Services [including Admission and Discharge Services, 99234-99236 as appropriate.]) | | |
|  | Procedure | UMLS:CPT:1013675 | Observation or Inpatient Care Services (Including Admission and Discharge Services) | | |
|  | Procedure | UMLS:CPT:99218 | Initial observation care, per day, for the evaluation and management of a patient which requires these 3 key components: A detailed or comprehensive history; A detailed or comprehensive examination; and Medical decision making that is straightforward or of low complexity. Counseling and/or coordination of care with other physicians, other qualified health care professionals, or agencies are provided consistent with the nature of the problem(s) and the patient's and/or family's needs. Usually, the problem(s) requiring admission to outpatient hospital "observation status" are of low severity. Typically, 30 minutes are spent at the bedside and on the patient's hospital floor or unit. | | |
|  | Procedure | UMLS:CPT:99219 | Initial observation care, per day, for the evaluation and management of a patient, which requires these 3 key components: A comprehensive history; A comprehensive examination; and Medical decision making of moderate complexity. Counseling and/or coordination of care with other physicians, other qualified health care professionals, or agencies are provided consistent with the nature of the problem(s) and the patient's and/or family's needs. Usually, the problem(s) requiring admission to outpatient hospital "observation status" are of moderate severity. Typically, 50 minutes are spent at the bedside and on the patient's hospital floor or unit. | | |
|  | Procedure | UMLS:CPT:99235 | Observation or inpatient hospital care, for the evaluation and management of a patient including admission and discharge on the same date, which requires these 3 key components: A comprehensive history; A comprehensive examination; and Medical decision making of moderate complexity. Counseling and/or coordination of care with other physicians, other qualified health care professionals, or agencies are provided consistent with the nature of the problem(s) and the patient's and/or family's needs. Usually the presenting problem(s) requiring admission are of moderate severity. Typically, 50 minutes are spent at the bedside and on the patient's hospital floor or unit. | | |
|  | Procedure | UMLS:CPT:99236 | Observation or inpatient hospital care, for the evaluation and management of a patient including admission and discharge on the same date, which requires these 3 key components: A comprehensive history; A comprehensive examination; and Medical decision making of high complexity. Counseling and/or coordination of care with other physicians, other qualified health care professionals, or agencies are provided consistent with the nature of the problem(s) and the patient's and/or family's needs. Usually the presenting problem(s) requiring admission are of high severity. Typically, 55 minutes are spent at the bedside and on the patient's hospital floor or unit. | | |
|  | Procedure | UMLS:CPT:99306 | Initial nursing facility care, per day, for the evaluation and management of a patient, which requires these 3 key components: A comprehensive history; A comprehensive examination; and Medical decision making of high complexity. Counseling and/or coordination of care with other physicians, other qualified health care professionals, or agencies are provided consistent with the nature of the problem(s) and the patient's and/or family's needs. Usually, the problem(s) requiring admission are of high severity. Typically, 45 minutes are spent at the bedside and on the patient's facility floor or unit. | | |
|  | Procedure | UMLS:CPT:99234 | Observation or inpatient hospital care, for the evaluation and management of a patient including admission and discharge on the same date, which requires these 3 key components: A detailed or comprehensive history; A detailed or comprehensive examination; and Medical decision making that is straightforward or of low complexity. Counseling and/or coordination of care with other physicians, other qualified health care professionals, or agencies are provided consistent with the nature of the problem(s) and the patient's and/or family's needs. Usually the presenting problem(s) requiring admission are of low severity. Typically, 40 minutes are spent at the bedside and on the patient's hospital floor or unit. | | |
|  | Procedure | UMLS:CPT:99305 | Initial nursing facility care, per day, for the evaluation and management of a patient, which requires these 3 key components: A comprehensive history; A comprehensive examination; and Medical decision making of moderate complexity. Counseling and/or coordination of care with other physicians, other qualified health care professionals, or agencies are provided consistent with the nature of the problem(s) and the patient's and/or family's needs. Usually, the problem(s) requiring admission are of moderate severity. Typically, 35 minutes are spent at the bedside and on the patient's facility floor or unit. | | |
|  | Procedure | UMLS:CPT:99304 | Initial nursing facility care, per day, for the evaluation and management of a patient, which requires these 3 key components: A detailed or comprehensive history; A detailed or comprehensive examination; and Medical decision making that is straightforward or of low complexity. Counseling and/or coordination of care with other physicians, other qualified health care professionals, or agencies are provided consistent with the nature of the problem(s) and the patient's and/or family's needs. Usually, the problem(s) requiring admission are of low severity. Typically, 25 minutes are spent at the bedside and on the patient's facility floor or unit. | | |
|  | Procedure | UMLS:SNOMED:305056002 | Admission procedure | | |
|  | Procedure | UMLS:SNOMED:32485007 | Hospital admission | | |
|  | Procedure | UMLS:SNOMED:50849002 | Emergency room admission | | |
|  | Procedure | UMLS:HCPCS:G8907 | Patient documented not to have experienced any of the following events: a burn prior to discharge; a fall within the facility; wrong site/side/patient/procedure/implant event; or a hospital transfer or hospital admission upon discharge from the facility | | |

| Dialysis | | | | |
| --- | --- | --- | --- | --- |
|  | **Outcome definition** | | | |
|  | | Procedure | UMLS:CPT:90935 | Hemodialysis procedure with single evaluation by a physician or other qualified health care professional |
|  | | Procedure | UMLS:CPT:90945 | Dialysis procedure other than hemodialysis (eg, peritoneal dialysis, hemofiltration, or other continuous renal replacement therapies), with single evaluation by a physician or other qualified health care professional |
|  | | Procedure | UMLS:CPT:90947 | Dialysis procedure other than hemodialysis (eg, peritoneal dialysis, hemofiltration, or other continuous renal replacement therapies) requiring repeated evaluations by a physician or other qualified health care professional, with or without substantial revision of dialysis prescription |
|  | | Procedure | UMLS:CPT:1006747 | Hemodialysis Access, Intervascular Cannulation for Extracorporeal Circulation, or Shunt Insertion Procedures on Arteries and Veins |
|  | | Procedure | UMLS:CPT:1012740 | Dialysis Services and Procedures |
|  | | Procedure | UMLS:CPT:1012752 | Hemodialysis Procedures |
|  | | Procedure | UMLS:CPT:1029674 | Dialysis Circuit Procedures |
|  | | Procedure | UMLS:SNOMED:302497006 | Hemodialysis |
|  | | Procedure | UMLS:ICD9CM:39.95 | Hemodialysis |
|  | | Diagnosis | UMLS:ICD10CM:Z99.2 | Dependence on renal dialysis |

| Kidney transplantation | | | | |
| --- | --- | --- | --- | --- |
|  | **Outcome definition** | | | |
|  | | Diagnosis | UMLS:ICD10CM:Z94.0 | Kidney transplant status |
|  | | Procedure | UMLS:ICD10PCS:0TY00Z0 | Transplantation of Right Kidney, Allogeneic, Open Approach |
|  | | Procedure | UMLS:ICD10PCS:0TY00Z1 | Transplantation of Right Kidney, Syngeneic, Open Approach |
|  | | Procedure | UMLS:ICD10PCS:0TY00Z2 | Transplantation of Right Kidney, Zooplastic, Open Approach |
|  | | Procedure | UMLS:ICD10PCS:0TY10Z0 | Transplantation of Left Kidney, Allogeneic, Open Approach |
|  | | Procedure | UMLS:ICD10PCS:0TY10Z1 | Transplantation of Left Kidney, Syngeneic, Open Approach |
|  | | Procedure | UMLS:ICD10PCS:0TY10Z2 | Transplantation of Left Kidney, Zooplastic, Open Approach |
|  | | Procedure | UMLS:SNOMED:70536003 | Transplant of kidney |
|  | | Procedure | UMLS:CPT:1008109 | Renal allotransplantation, implantation of graft |

| Skin cancer | | | | |
| --- | --- | --- | --- | --- |
|  | **Outcome definition** | | | |
|  | | Diagnosis | UMLS:ICD10CM:C43-C44 | Melanoma and other malignant neoplasms of skin |

| Herniated disc | | | | |
| --- | --- | --- | --- | --- |
| Outcome definition | | | |  |
|  | Diagnosis | UMLS:ICD10CM:M50 | Cervical disc disorders | |
|  | Diagnosis | UMLS:ICD10CM:M51 | Thoracic, thoracolumbar, and lumbosacral intervertebral disc disorders | |

| Hemorrhoids | | | | |
| --- | --- | --- | --- | --- |
|  | **Outcome definition** | | | |
|  | | Diagnosis | UMLS:ICD10CM:K64 | Hemorrhoids and perianal venous thrombosis |

| Upper respiratory infections | | | | |
| --- | --- | --- | --- | --- |
|  | **Outcome definition** | | | |
|  | | Diagnosis | UMLS:ICD10CM:J00-J06 | Acute upper respiratory infections |

| COPD | | | | |
| --- | --- | --- | --- | --- |
| Outcome definition | | | |  |
|  | Diagnosis | UMLS:ICD10CM:J44 | Other chronic obstructive pulmonary disease | |

| Gastroesophageal reflux disease | | | | |
| --- | --- | --- | --- | --- |
|  | **Outcome definition** | | | |
|  | | Diagnosis | UMLS:ICD10CM:K21 | Gastro-esophageal reflux disease |

**Table S1. Numbers and Demographics of Individuals Excluded Because of a Lack of Any Follow-Up**

| Variables | No follow-up, No (%) | |
| --- | --- | --- |
|  | SGLT-2i users | Non-users |
| Number of patients | 10 | 10 |
| Demographics | | |
| Age, mean (SD), y | 61.6 (2.72) | 64.7 (2.5) |
| Male | 10 (100%) | 6 (60%) |
| Black or African American | 0 (0%) | 2 (20%) |
| Asian | 10 (100%) | 1 (10%) |
| Hispanic or Latino | 0 (0%) | 3 (30%) |
| White | 0 (0%) | 5 (50%) |

Abbreviations: SGLT2-2i, sodium–glucose cotransporter 2 inhibitor

**Table S2. Presumptive causes of dialysis**

| **Before PSM** | **SGLT-2i users**  **(n=781)** | **Non-users**  **(n=48,981)** | **P-value** |
| --- | --- | --- | --- |
| advanced CKD | 186 (23.8%) | 15276 (31.2%) | <0.0001 |
| AKI | 595 (76.2%) | 33705 (68.8%) | <0.0001 |
| Cardiogenic/ hypovolemic shock | 62 (7.9%) | 2373 (4.8%) | 0.9999 |
| Sepsis | 183 (23.4%) | 11446 (23.4%) | 0.9999 |
| Hepatorenal syndrome | 21 (2.7%) | 1203 (2.5%) | 0.7329 |
| Obstructive uropathy | 15 (1.9%) | 1670 (3.4%) | 0.0319 |
| Heart failure | 290 (37.1%) | 13516 (27.6%) | <0.0001 |
| Others* | 25 (3.2%) | 3498 (7.1%) | <0.0001 |

| **After PSM** | **SGLT-2i users**  **(n=771)** | **Non-users**  **(n=771)** | **P-value** |
| --- | --- | --- | --- |
| advanced CKD | 184 (23.9%) | 187 (24.3%) | 0.9051 |
| AKI | 587 (76.1%) | 584 (75.7%) | 0.9051 |
| Cardiogenic/ hypovolemic shock | 61 (7.9%) | 62 (8.0%) | 0.9999 |
| Sepsis | 180 (23.3%) | 177 (23.0%) | 0.9038 |
| Hepatorenal syndrome | 21 (2.7%) | 18 (2.3%) | 0.7456 |
| Obstructive uropathy | 15 (2.0%) | 16 (2.1%) | 0.9999 |
| Heart failure | 285 (37.0%) | 286 (37.1%) | 0.9999 |
| Others* | 25 (3.2%) | 26 (3.3%) | 0.9999 |

*Others: hypertension crisis, drug-related AKI, Contrast nephropathy, etc. Advanced CKD defined as baseline kidney function less than eGFR 15 ml/min/1.73^2^. Abbreviation: AKI, acute kidney injury; CKD, chronic kidney disease; PSM, propensity score matching; SGLT-2i, sodium–glucose cotransporter 2 inhibitor

**Table S3. Incidence rate and E-values of primary outcomes among SGLT-2i users compared to non-users after prosperity score matching**

| **Outcome** | **Total** | **Event** | **aHR (95%CI)** | **E-value (upper limit of CI) for aHR** |
| --- | --- | --- | --- | --- |
| **All-cause mortality** | 1542 | 169 | 0.485 (0.341,0.69) | 3.54 (2.26) |
| SGLT-2i users | 771 | 42 |  |  |
| Non-users | 771 | 127 |  |  |
| **MACE** | 928 | 145 | 0.516 (0.357,0.748) | 3.29 (2.01) |
| SGLT-2i users | 456 | 39 |  |  |
| Non-users | 472 | 106 |  |  |

Abbreviations: aHR, adjust hazard ratio; CI, confidence interval; MACE; major adverse cardiovascular event; SGLT-2i, sodium–glucose cotransporter 2 inhibitor

**Table S4. Risk of mortality in patients with T2DM at dialysis commencement: comparison between SGLT-2i users and non-users after propensity score matching**

The table presents results from a cohort analysis after propensity score matching. Cohort 1 consist of patients with T2DM at dialysis commencement who used SGLT-2is, while cohort 2 comprises those who did not use of SGLT-2is.

| **All-cause mortality** | | | | | | | | | | | | |
| --- | --- | --- | --- | --- | --- | --- | --- | --- | --- | --- | --- | --- |
|  | | **Risk analysis** | | | | | | | | | | |
|  |  | | | Cohort | | | Patients in cohort | Patients with outcome | Risk | | | |
|  | | |  | 1 | | SGLT-2i users | 771 | 42 | 0.054 | | | |
|  | | |  | 2 | | Non-users | 771 | 127 | 0.165 | | | |
|  | | | | | | | | | | | | |
|  | | |  |  | | |  | 95% CI | z | p |  |  |
|  | | |  | **Risk Difference** | | | -0.110 | (-0.141, -0.080) | -6.929 | 0.000 |  |  |
|  | | |  | **Risk Ratio** | | | 0.331 | (0.237, 0.462) | N/A | N/A |  |  |
|  | | |  | **Odds Ratio** | | | 0.292 | (0.203, 0.421) | N/A | N/A |  |  |
|  | | | | | | | | | | | | |
|  | |  | | | 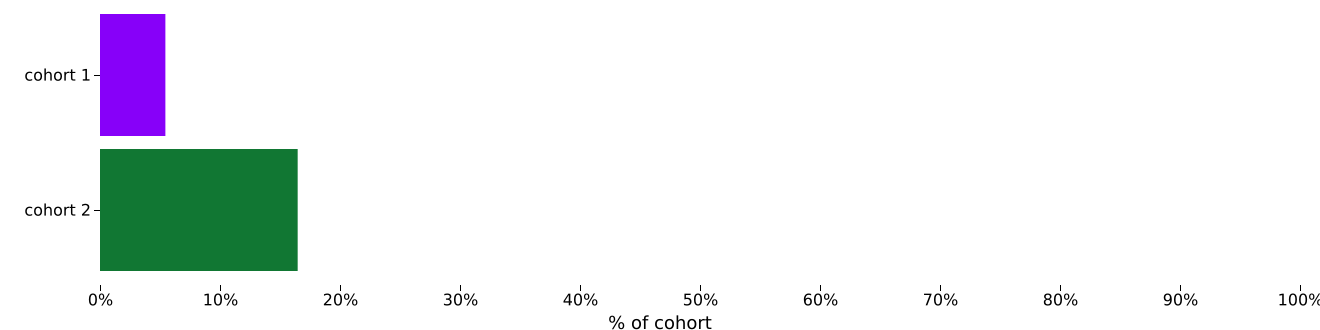 | | | | | | | |
|  | | **Kaplan - Meier survival analysis** | | | | | | | | | | |
|  | | |  | Cohort | | | Patients in cohort | Patients with outcome | Median survival (days) | Survival probability at end of time window | | |
|  | | |  | 1 | | SGLT-2i users | 771 | 42 | -- | 88.82% | | |
|  | | |  | 2 | | Non-users | 771 | 127 | -- | 74.35% | | |
|  | | | | | | | | | | | | |
|  | | |  |  | | | χ^2^ | df | p |  |  |  |
|  | | |  | **Log-Rank Test** | | | 16.874 | 1 | 0.000 |  |  |  |
|  | | | | | | | | | | | | |
|  | | |  |  | | | Hazard Ratio | 95% CI | χ^2^ | df | p | |
|  | | |  | **Hazard Ratio and Proportionality** | | | 0.485 | (0.341, 0.690) | 1.939 | 1 | 0.164 | |
|  | | | | | | | | | | | | |
|  | |  | | | 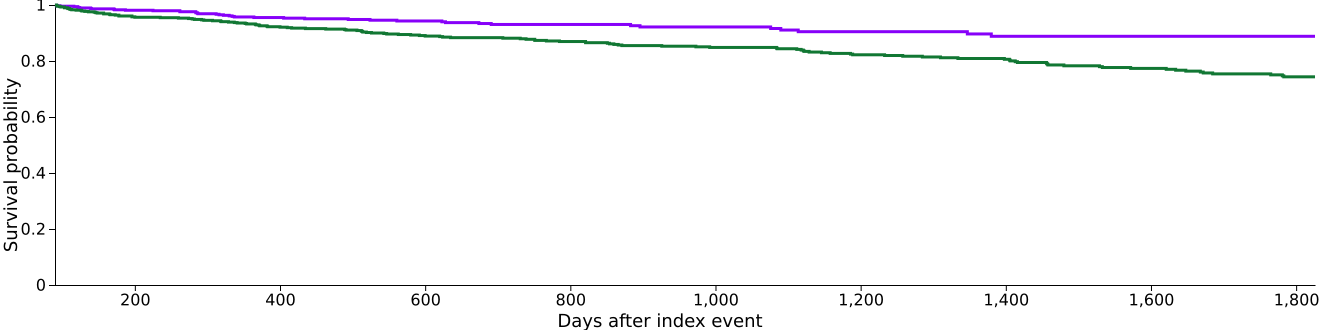 | | | | | | | |
|  | | **Number of instances** | | | | | | | | | | |
|  | | |  | Cohort | | | Patients in cohort | Patients with outcome | Mean | Standard Deviation | Median | |
|  | | |  | 1 | | SGLT-2i users | 771 | 42 | 1.071 | 0.342 | 1 | |
|  | | |  | 2 | | Non-users | 771 | 127 | 1.331 | 2.226 | 1 | |
|  | | | | | | | | | | | | |
|  | | |  |  | | | t | df | p |  |  |  |
|  | | |  | **Test Statistics** | | | -0.751 | 167 | 0.454 |  |  |  |
|  | | | | | | | | | | | | |
|  | |  | | | 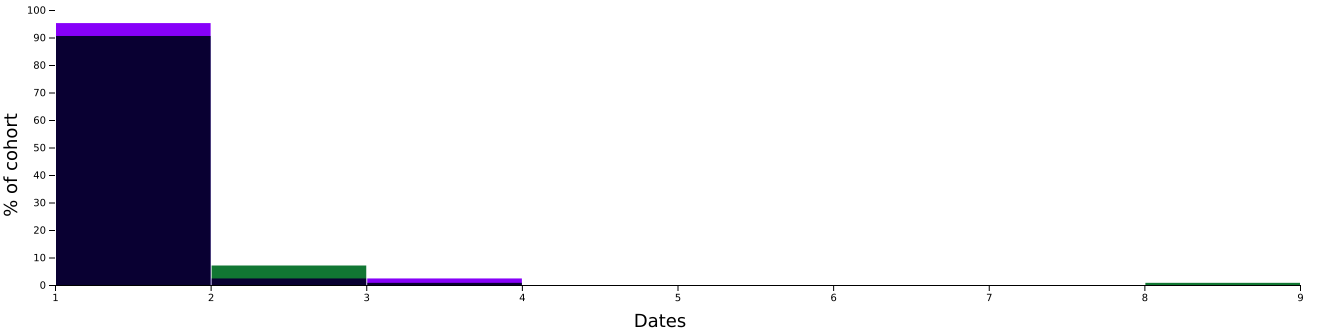 | | | | | | | |
|  | |  | | | 0 data points for Cohort 1 and 1 data points for Cohort 2 were omitted for display purposes. | | | | | | | |

**Table S5. Risk of MACE in patients with T2DM at dialysis commencement: comparison between SGLT-2i users and non-users after propensity score matching.**

The table presents results from a cohort analysis after propensity score matching. Patients with a history of MACE were part of the baseline cohort but were excluded from this analysis. Cohort 1 consist of patients with T2DM at dialysis commencement who used SGLT-2is, while cohort 2 comprises those who did not use of SGLT-2is.

| **MACE** | | | | | | | | | | | | |
| --- | --- | --- | --- | --- | --- | --- | --- | --- | --- | --- | --- | --- |
|  | | **Risk analysis excluding patients with outcome prior to the time window** | | | | | | | | | | |
|  |  | | | Cohort | | | Patients in cohort | Patients with outcome | Risk | | | |
|  | | |  | 1 | | SGLT-2i users | 456 | 39 | 0.086 | | | |
|  | | |  | 2 | | Non-users | 472 | 106 | 0.225 | | | |
|  | | | | | | | | | | | | |
|  | | |  |  | | |  | 95% CI | z | p |  |  |
|  | | |  | **Risk Difference** | | | -0.139 | (-0.185, -0.093) | -5.832 | 0.000 |  |  |
|  | | |  | **Risk Ratio** | | | 0.381 | (0.270, 0.537) | N/A | N/A |  |  |
|  | | |  | **Odds Ratio** | | | 0.323 | (0.218, 0.478) | N/A | N/A |  |  |
|  | | | | | | | | | | | | |
|  | |  | | | 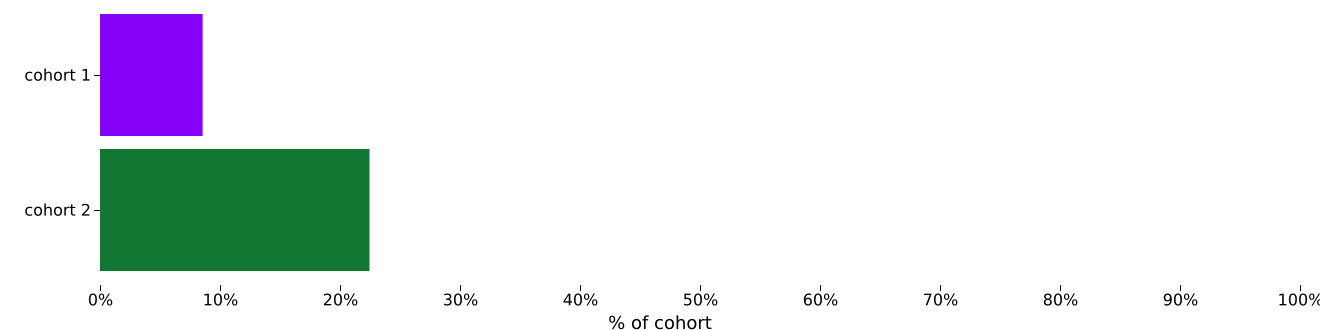 | | | | | | | |
|  | |  | | | 315 patients in Cohort 1 and 299 patients in Cohort 2 were excluded from results because they had the outcome prior to the time window. | | | | | | | |
|  | | **Kaplan - Meier survival analysis excluding patients with outcome prior to the time window** | | | | | | | | | | |
|  | | |  | Cohort | | | Patients in cohort | Patients with outcome | Median survival (days) | Survival probability at end of time window | | |
|  | | |  | 1 | | SGLT-2i users | 456 | 39 | -- | 80.33% | | |
|  | | |  | 2 | | Non-users | 472 | 106 | -- | 66.17% | | |
|  | | | | | | | | | | | | |
|  | | |  |  | | | χ^2^ | df | p |  |  |  |
|  | | |  | **Log-Rank Test** | | | 12.694 | 1 | 0.000 |  |  |  |
|  | | | | | | | | | | | | |
|  | | |  |  | | | Hazard Ratio | 95% CI | χ^2^ | df | p | |
|  | | |  | **Hazard Ratio and Proportionality** | | | 0.516 | (0.357, 0.748) | 0.002 | 1 | 0.962 | |
|  | | | | | | | | | | | | |
|  | |  | | | 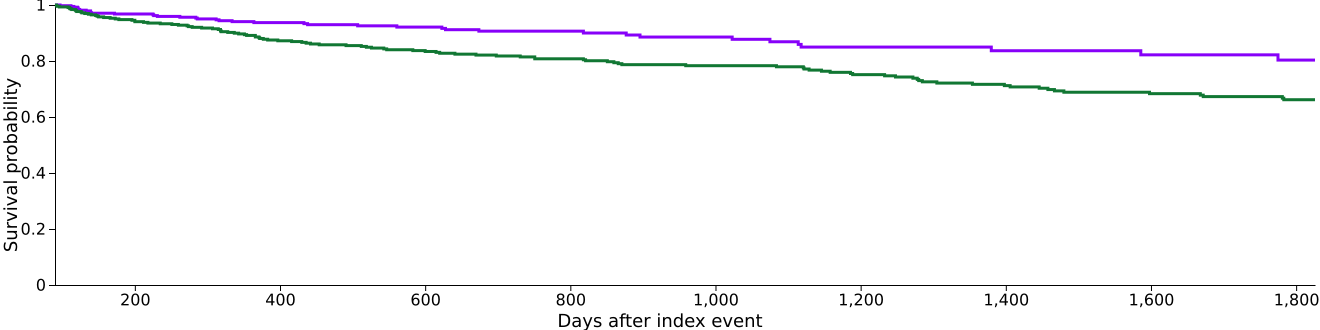 | | | | | | | |
|  | |  | | | 315 patients in Cohort 1 and 299 patients in Cohort 2 were excluded from results because they had the outcome prior to the time window. | | | | | | | |
|  | | **Number of instances excluding patients with outcome prior to the time window** | | | | | | | | | | |
|  | | |  | Cohort | | | Patients in cohort | Patients with outcome | Mean | Standard Deviation | Median | |
|  | | |  | 1 | | SGLT-2i users | 456 | 39 | 1.769 | 1.677 | 1 | |
|  | | |  | 2 | | Non-users | 472 | 106 | 3.745 | 8.368 | 1 | |
|  | | | | | | | | | | | | |
|  | | |  |  | | | t | df | p |  |  |  |
|  | | |  | **Test Statistics** | | | -1.461 | 143 | 0.146 |  |  |  |
|  | | | | | | | | | | | | |
|  | |  | | | 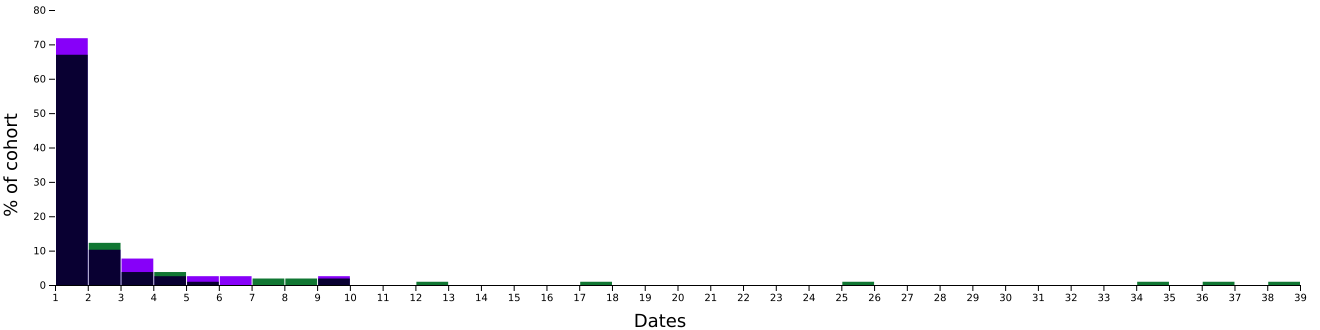 | | | | | | | |
|  | |  | | | 0 data points for Cohort 1 and 1 data points for Cohort 2 were omitted for display purposes. 315 patients in Cohort 1 and 299 patients in Cohort 2 were excluded from results because they had the outcome prior to the time window. | | | | | | | |

**Table S6. Incidence Rate of MACE Subcomponents Among SGLT-2i Users Compared to Non-Users After Propensity Score Matching**

|  | **Patients with outcome** | | | | | **aHR (95%CI)** | | | | | |  |  |  |  |
| --- | --- | --- | --- | --- | --- | --- | --- | --- | --- | --- | --- | --- | --- | --- | --- |
|  | **SGLT-2i users** | | | | **Non-users** | |  | | |  |  |  |  |  |  |
| **After PSM** | |  | |  | | | |  | | | | | |  |  |
| MACE | | 8.6% (39/456) | | 22.5% (106/472) | | | | 0.516(0.357,0.748) | | | | | |  |  |
| Cardiac arrest/ mortality | | 4.5% (20/456) | | 14.3% (68/472) | | | | 0.47(0.325,0.68) | | | | | |  |  |
| Cerebrovascular accident | | 2.5% (12/456) | | 4.4% (21/472) | | | | 0.951(0.534,1.693) | | | | | |  |  |
| AMI/ unstable angina | | | 1.5% (7/456) | 3.7% (17/472) | | | | | 0.547(0.253,1.183) | | | | | |  |

Abbreviations: aHR, adjust hazard ratio; CI, confidence interval; MACE; major adverse cardiovascular event; PSM, propensity score matching; SGLT-2i, sodium–glucose cotransporter 2 inhibitor

**Table S7. Incidence rate of among SGLT-2i users compared to non-users after prosperity score matching**

|  | **Outcome** | | **Patients with outcome** | | | **aHR (95%CI)** | | | | | |  | |
| --- | --- | --- | --- | --- | --- | --- | --- | --- | --- | --- | --- | --- | --- |
|  |  | | **SGLT-2i users** | **Non-users** | | |  | |  |  |  |  |  |
| Ketoacidosis | | 9.9% (39/392) | | 11.7% (53/452) | | | 1.25(0.82-1.90) | | | |  | |  |
| UTI/Genital infection | | 5.7% (32/560) | | 7.8% (44/565) | | | 1.03(0.65-1.63) | | | |  | |  |
| 3P-MACE | | 9.0% (45/501) | | 21.7% (117/538) | | | 0.60(0.42-0.85) | | | |  | |  |
| Hypoglycemia | | 3.5% (22/621) | | 4.9% (29/596) | | | 0.95(0.55-1.67) | | | |  | |  |
| Dehydration | | 1.9% (13/697) | | 3.6% (26/713) | | | 0.77(0.39-1.52) | | | |  | |  |
| Fracture | | 2.4% (16/674) | | 4.5% (31/688) | | | 0.86(0.47-1.58) | | | |  | |  |
| BKA | | 1.3% (10/743) | | 1.3% (10/745) | | | 0.71(0.18-2.90) | | | |  | |  |
| 90-day Readmission | | | 4.5% (35/771) | | 4.8% (27/771) | | | 0.82 (0.68-0.99) | | | | |  |

Abbreviations: aHR, adjust hazard ratio; BKA, below-knee amputation; CI, confidence interval; 3P-MACE; 3-piont major adverse cardiovascular event; SGLT-2i, sodium–glucose cotransporter 2 inhibitor; UTI, urinary tract infection

**Table S8. Landmark analysis of SGLT-2 Inhibitors in T2DM Patients after Dialysis Initiation: Focus on Kidney Outcomes**

|  | **SGLT-2i users (total/event)** | **Non-users (total/event)** | **aHR (95%CI)** | **P-value** |
| --- | --- | --- | --- | --- |
| **D0-D90** |  |  |  |  |
| Dialysis | 771/25 | 771/60 | 0.448 (0.281,0.714) | 0.0005 |
| Kidney Transplantation | 771/<10 | 771/0 | N/A | N/A |
| Combined mortality/remaining on dialysis | 771/38 | 771/81 | 0.493 (0.335,0.725) | 0.0002 |
| **D180-D270** |  |  |  |  |
| Dialysis | 771/20 | 771/56 | 0.41 (0.246,0.683) | 0.0004 |
| Kidney Transplantation | 771/<10 | 771/0 | N/A | N/A |
| Combined mortality/remaining on dialysis | 771/27 | 771/65 | 0.488 (0.314,0.759) | 0.0011 |

Abbreviations: aHR, adjusted hazard ratio; CI, confidence interval; SGLT-2i, sodium–glucose cotransporter 2 inhibitor

# The index date was set at 90 days after dialysis commencement.

**Table S9. Incidence of pre-specified outcomes with different follow-up time between SGLT-2i Users and Non-users**

|  | **SGLT-2i users** | **Non-users** | **aHR** | **95%CI** |
| --- | --- | --- | --- | --- |
| All-cause mortality | (n=771) | (n=771) |  |  |
| 1 year | 3.6% (28) | 6.0% (46) | 0.678 | (0.421,1.093) |
| 2 years | 7.8% (37) | 11.4% (88) | 0.503 | (0.341,0.743) |
| 3 years | 5.2% (40) | 12.0% (93) | 0.591 | (0.407,0.858) |
| 4 years | 5.3% (41) | 14.9% (115) | 0.561 | (0.396,0.794) |
| 5 years | 5.4% (42) | 16.5% (127) | 0.485 | (0.341,0.69) |
| MACE | (n=456) | (n=472) |  |  |
| 1 years | 4.9% (22) | 7.1% (34) | 0.785 | (0.459,1.342) |
| 2 years | 6.4% (29) | 12.1% (57) | 0.606 | (0.389,0.943) |
| 3 years | 7.5% (35) | 17.2% (81) | 0.569 | (0.38,0.852) |
| 4 years | 8.1% (37) | 18.6% (88) | 0.627 | (0.425,0.927) |
| 5 years | 8.6% (39) | 22.5% (106) | 0.516 | (0.357,0.748) |

Abbreviation: aHR, adjusted hazard ratio; CI, confidence interval; MACE, major adverse cardiovascular events; SGLT-2i, sodium–glucose cotransporter 2 inhibitors

**Table S10. Landmark analysis for primary outcomes across varied durations of SGLT-2is use**

|  | **aHR (95%CI)** | **P-value** |
| --- | --- | --- |
| **Usage of SGLT-2is within a 14-Day Duration** |  |  |
| All-cause mortality | 0.415 (0.233,0.74) | 0.0021 |
| MACE | 0.591 (0.33,1.06) | 0.0744 |
| **Usage of SGLT-2is within a 30-Day Duration** |  |  |
| All-cause mortality | 0.447 (0.285,0.702) | 0.0003 |
| MACE | 0.606 (0.383,0.961) | 0.0314 |
| **Usage of SGLT-2is within a 60-Day Duration** |  |  |
| All-cause mortality | 0.48 (0.332,0.694) | <0.0001 |
| MACE | 0.586 (0.385,0.891) | 0.0114 |

Abbreviations: aHR, adjusted hazard ratio; CI, confidence interval; MACE, major adverse cardiovascular events; SGLT-2is, sodium–glucose cotransporter 2 inhibitor

# landmark analysis refers to the renal replacement therapy a time point occurring during the follow-up period (known as the landmark time) and analyzing only those subjects who have dialyzed until the landmark time. [5, 6]

**Table S11. Sensitivity Analysis for All-Cause Mortality Between SGLT-2 is Users and Non-users**

|  | **aHR (95%CI)** |
| --- | --- |
| **All eligible subjects without weighting** |  |
| Original model | 0.454 (0.335,0.615) |
| **Eligible subjects with different exclusion criteria, 1:1 PSM** |  |
| Exclude died within 2 months after dialysis | 0.448 (0.321,0.626) |
| Exclude died within 1 month after dialysis | 0.548 (0.39,0.768) |
| Include all patients after dialysis | 0.421 (0.297,0.596) |
| **Cox regression models with different covariates** |  |
| Model 1 (age and gender, ethnicity) | 0.458 (0.341,0.616) |
| Model 2 (age, gender, ethnicity, comorbidities) | 0.465 (0.346,0.626) |
| Model 3 (age, gender, ethnicity, comorbidities, K, eGFR, HbA1c) | 0.608 (0.428,0.864) |
| Model 4 (age, gender, ethnicity, comorbidities, K, eGFR, HbA1c hyperlipidemia) | 0.537 (0.38,0.759) |
| Model 5 (age, gender, ethnicity, comorbidities, K, eGFR, HbA1c hyperlipidemia, use of ACEI/ARB) | 0.497 (0.352,0.7) |
| Model 6 (age, gender, ethnicity, comorbidities, K, eGFR, HbA1c hyperlipidemia, use of ACEI/ARB and Beta-blocker) | 0.498 (0.354,0.702) |
| Model 7 (age, gender, ethnicity, comorbidities, K, eGFR, HbA1c hyperlipidemia, use of ACEI/ARB, Beta-blocker, and GLP-1 agonists) | 0.559 (0.395,0.791) |
| Model 8 (final full model) | 0.485 (0.341,0.69) |

Abbreviations: ACEI, angiotensin converting enzyme inhibitors; aHR, adjusted hazard ratio; ARB, CI, confidence interval; eGFR, estimated Glomerular filtration rate; GLP-1; glucagon-like peptide 1; HbA1c, glycated hemoglobin; PSM, propensity score matching; SGLT2-2is, sodium–glucose cotransporter 2 inhibitor

**Table S12. Sensitivity Analysis for MACE Between SGLT-2is Users and Non-users**

|  | **aHR (95%CI)** |
| --- | --- |
| **All eligible subjects without weighting** |  |
| Original model | 0.559 (0.408,0.765) |
| **Eligible subjects with different exclusion criteria, 1:1 PSM** |  |
| Exclude died within 2 months after dialysis | 0.58 (0.402,0.836) |
| Exclude died within 1 month after dialysis | 0.582 (0.402,0.844) |
| Include all patients after dialysis | 0.526 (0.358,0.772) |
| **Cox regression models with different covariates** |  |
| Model 1 (age and gender, ethnicity) | 0.526 (0.383,0.724) |
| Model 2 (age, gender, ethnicity, comorbidities) | 0.535 (0.389,0.736) |
| Model 3 (age, gender, ethnicity, comorbidities, K, eGFR, HbA1c) | 0.627 (0.43,0.913) |
| Model 4 (age, gender, ethnicity, comorbidities, K, eGFR, HbA1c hyperlipidemia) | 0.595 (0.409,0.867) |
| Model 5 (age, gender, ethnicity, comorbidities, K, eGFR, HbA1c hyperlipidemia, use of ACEI/ARB) | 0.656 (0.447,0.961) |
| Model 6 (age, gender, ethnicity, comorbidities, K, eGFR, HbA1c hyperlipidemia, use of ACEI/ARB and Beta-blocker) | 0.561 (0.387,0.815) |
| Model 7 (age, gender, ethnicity, comorbidities, K, eGFR, HbA1c hyperlipidemia, use of ACEI/ARB, Beta-blocker, and GLP-1 agonists) | 0.713 (0.485, 0.985) |
| Model 8 (final full model) | 0.516 (0.357,0.748) |

Abbreviations: ACEI, angiotensin converting enzyme inhibitors; aHR, adjusted hazard ratio; ARB, CI, confidence interval; eGFR, estimated Glomerular filtration rate; GLP-1; glucagon-like peptide 1; HbA1c, glycated hemoglobin; PSM, propensity score matching; MACE, major adverse cardiac event; SGLT2-2is, sodium–glucose cotransporter 2 inhibitors

**Table S13. Sensitivity Analysis of SGLT-2 Inhibitors usage Status After Index Date**

|  | **SGLT-2i users (total/event)** | **Non-users (total/event)** | **aHR (95%CI)** | **P-value** |
| --- | --- | --- | --- | --- |
| **Continued Usage 1-3 months after index date** |  |  |  |  |
| All-cause mortality | 246/10 | 246/49 | 0.245 (0.12,0.503) | < 0.0001 |
| MACE | 158/13 | 138/35 | 0.397 (0.206,0.767) | 0.0044 |
| **Discontinued Usage 1-3 months after index date** |  |  |  |  |
| All-cause mortality | 525/31 | 525/78 | 0.559 (0.365,0.856) | 0.0067 |
| MACE | 298/26 | 334/71 | 0.603 (0.375,0.97) | 0.035 |

Abbreviations: aHR, adjusted hazard ratio; CI, confidence interval; MACE, major adverse cardiovascular events; SGLT-2i, sodium–glucose cotransporter 2 inhibitor

**Figure S1.** **Comparison of the pre-specified outcomes of patients treated with SGLT-2is versus those non-users before prosperity score matching.**

**
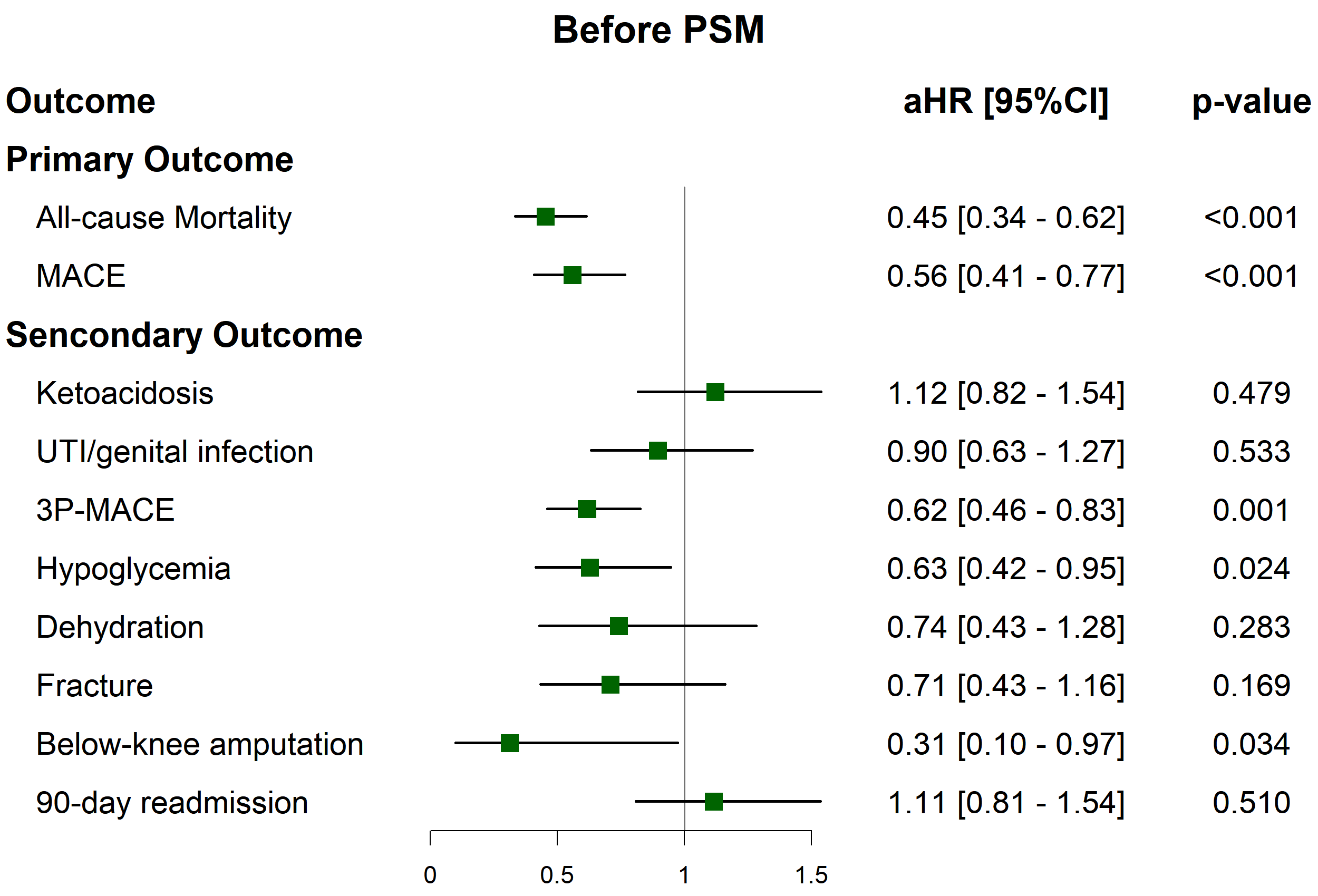
**

The forest plots illustrated the adjusted HRs of all-cause mortality, MACE, and other secondary outcomes for SGLT-2is users versus non-users before and after propensity score matching. The plots present both the adjusted HRs and their 95% confidence intervals (CIs), represented as error bars. The vertical line denotes an aHR of 1.00, with lower limits of the 95% CIs exceeding 1.00 indicating a statistically significant increased risk. **Abbreviations:** aHR, adjust hazard ratio; 3p-MACE, 3-piont major adverse cardiac event; MACE, major adverse cardiac event; PS, propensity score; UTI, urinary tract infection

**Figure S2.** **Negative outcome, positive and negative exposure controls**

**
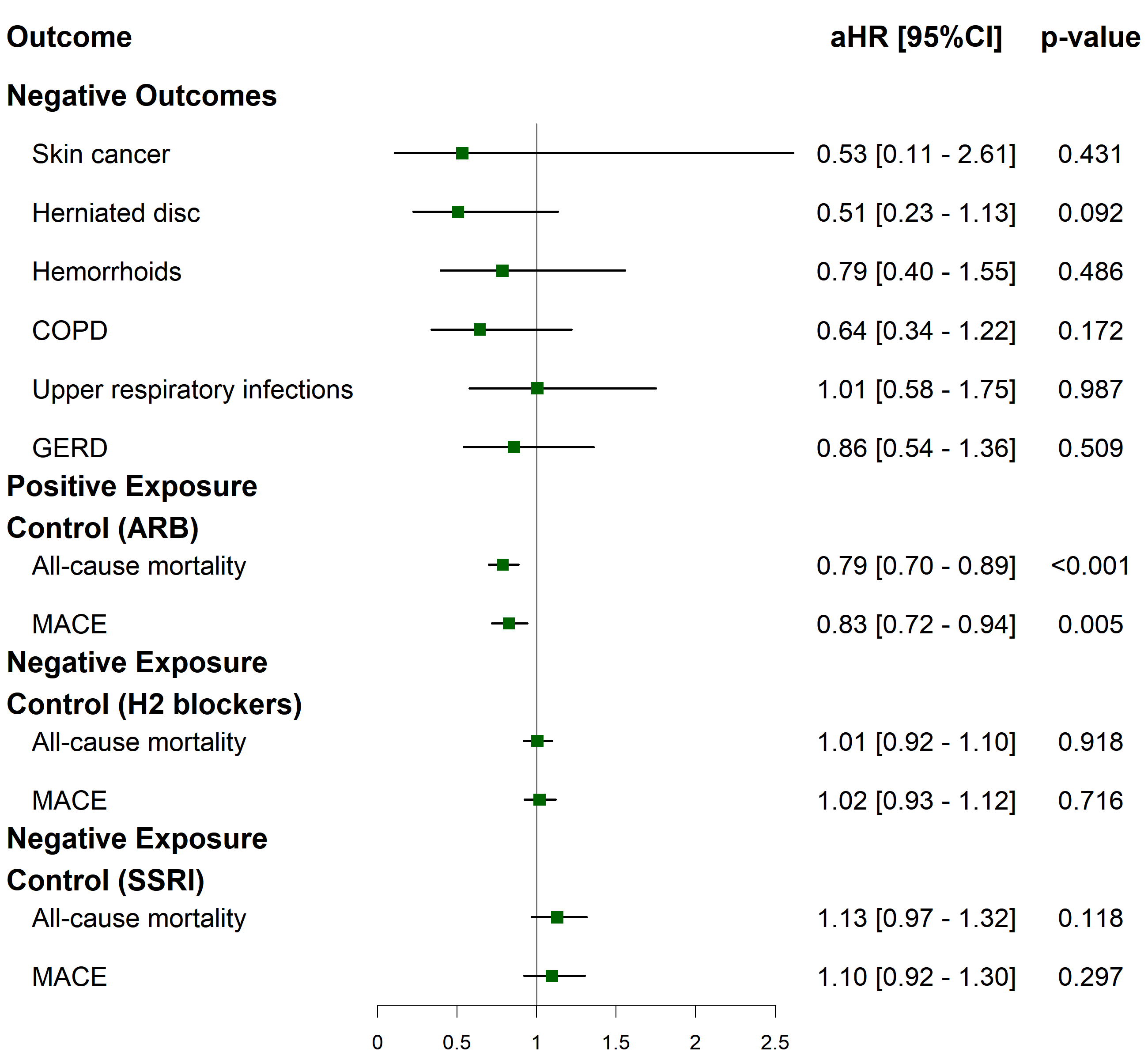
**

**Abbreviations:** aHR, adjust hazard ratio; ARB, angiotensin Ⅱ receptor blocker; COPD, chronic obstructive pulmonary diseases; GERD, gastroesophageal reflux disease; GLP-1 analogue, glucagon-like peptide-1 analogue; H2 blockers, histamine type2 receptor antagonists; MACE, major adverse cardiac event; SSRI, selective serotonin reuptake inhibitor

**Figure S3.** **Sensitivity study on various types and dosages of SGLT-2is**

**
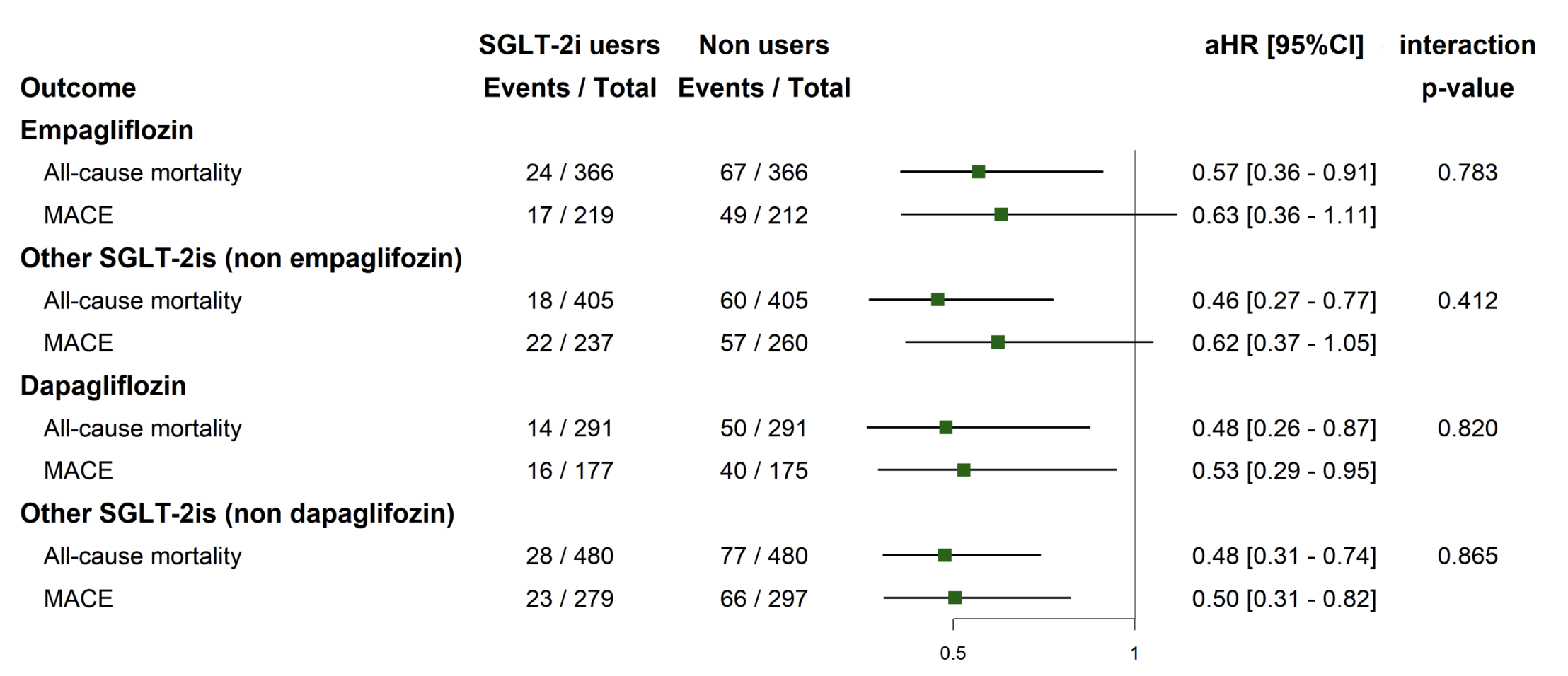
**

**Abbreviations:** aHR, adjust hazard ratio; MACE, major adverse cardiac event; SGLT-2is, sodium–glucose cotransporter 2 inhibitors

**Supplementary reference**

[1] Pan H-C, Chen J-Y, Chen H-Y, et al. (2024) Sodium-Glucose Cotransport Protein 2 Inhibitors in Patients With Type 2 Diabetes and Acute Kidney Disease. JAMA Network Open 7(1): e2350050-e2350050. 10.1001/jamanetworkopen.2023.50050

[2] Wu VC, Chen JY, Lin YH, Wang CY, Lai CC (2023) Assessing the cardiovascular events and clinical outcomes of COVID-19 on patients with primary aldosteronism. J Microbiol Immunol Infect. 10.1016/j.jmii.2023.09.005

[3] Topaloglu U, Palchuk MB (2018) Using a Federated Network of Real-World Data to Optimize Clinical Trials Operations. JCO Clin Cancer Inform 2: 1-10. 10.1200/cci.17.00067

[4] MacKenzie SL, Wyatt MC, Schuff R, Tenenbaum JD, Anderson N (2012) Practices and perspectives on building integrated data repositories: results from a 2010 CTSA survey. J Am Med Inform Assoc 19(e1): e119-124. 10.1136/amiajnl-2011-000508

[5] Anderson JR, Cain KC, Gelber RD (1983) Analysis of survival by tumor response. J Clin Oncol 1(11): 710-719. 10.1200/JCO.1983.1.11.710

[6] Buyse M, Piedbois P (1996) On the relationship between response to treatment and survival time. Stat Med 15(24): 2797-2812. 10.1002/(sici)1097-0258(19961230)15:24<2797::Aid-sim290>3.0.Co;2-v

**STROBE Statement—checklist of items that should be included in reports of observational studies**

|  | | Item No | Recommendation | Page No |  |
| --- | --- | --- | --- | --- | --- |
| **Title and abstract** | | 1 | (*a*) Indicate the study’s design with a commonly used term in the title or the abstract | P.1 |  |
|  |  |  | (*b*) Provide in the abstract an informative and balanced summary of what was done and what was found | P.4-5 |  |
| Introduction | | | |  |  |
| Background/rationale | | 2 | Explain the scientific background and rationale for the investigation being reported | P.6-8 |  |
| Objectives | | 3 | State specific objectives, including any prespecified hypotheses | P.7-8 |  |
| Methods | | | |  |  |
| Study design | | 4 | Present key elements of study design early in the paper | P.10-11 |  |
| Setting | | 5 | Describe the setting, locations, and relevant dates, including periods of recruitment, exposure, follow-up, and data collection | P.9-10,12 |  |
| Participants | | 6 | (*a*) *Cohort study*—Give the eligibility criteria, and the sources and methods of selection of participants. Describe methods of follow-up  *Case-control study*—Give the eligibility criteria, and the sources and methods of case ascertainment and control selection. Give the rationale for the choice of cases and controls  *Cross-sectional study*—Give the eligibility criteria, and the sources and methods of selection of participants | P.10,11,15  Figure.1, Table S1 |  |
|  |  |  | (*b*) *Cohort study*—For matched studies, give matching criteria and number of exposed and unexposed  *Case-control study*—For matched studies, give matching criteria and the number of controls per case | P. 10,11,15  Figure.1,  Table S1 |  |
| Variables | | 7 | Clearly define all outcomes, exposures, predictors, potential confounders, and effect modifiers. Give diagnostic criteria, if applicable | P12-14  Suppl. Method |  |
| Data sources/ measurement | | 8* | For each variable of interest, give sources of data and details of methods of assessment (measurement). Describe comparability of assessment methods if there is more than one group | P. 9-10 |  |
| Bias | | 9 | Describe any efforts to address potential sources of bias | P.14-16 |  |
| Study size | | 10 | Explain how the study size was arrived at | Figure 1 |  |
| Quantitative variables | | 11 | Explain how quantitative variables were handled in the analyses. If applicable, describe which groupings were chosen and why | P.12-14 |  |
| Statistical methods | | 12 | (*a*) Describe all statistical methods, including those used to control for confounding | P.15-17 |  |
|  |  |  | (*b*) Describe any methods used to examine subgroups and interactions | P.14 |  |
|  |  |  | (*c*) Explain how missing data were addressed | P.16 |  |
|  |  |  | (*d*) *Cohort study*—If applicable, explain how loss to follow-up was addressed  *Case-control study*—If applicable, explain how matching of cases and controls was addressed  *Cross-sectional study*—If applicable, describe analytical methods taking account of sampling strategy | P.16 |  |
|  |  |  | (*e*) Describe any sensitivity analyses | P.14-15 |  |
| Results | | | |  | |
| Participants | 13* | (a) Report numbers of individuals at each stage of study—eg numbers potentially eligible, examined for eligibility, confirmed eligible, included in the study, completing follow-up, and analysed | | P.18-19  Figure 1 | |
|  |  | (b) Give reasons for non-participation at each stage | | P.10-11  Figure.1 | |
|  |  | (c) Consider use of a flow diagram | | Figure.1 | |
| Descriptive data | 14* | (a) Give characteristics of study participants (eg demographic, clinical, social) and information on exposures and potential confounders | | P.18-19  Table. 1 | |
|  |  | (b) Indicate number of participants with missing data for each variable of interest | | P.18  Table. S1 | |
|  |  | (c) *Cohort study*—Summarise follow-up time (eg, average and total amount) | | P.18 | |
| Outcome data | 15* | *Cohort study*—Report numbers of outcome events or summary measures over time  *Case-control study—*Report numbers in each exposure category, or summary measures of exposure  *Cross-sectional study—*Report numbers of outcome events or summary measures | | P.19,20, Table. S3-7 | |
| Main results | 16 | (*a*) Give unadjusted estimates and, if applicable, confounder-adjusted estimates and their precision (eg, 95% confidence interval). Make clear which confounders were adjusted for and why they were included | | Figure. S1 | |
|  |  | (*b*) Report category boundaries when continuous variables were categorized | | Table.1 | |
|  |  | (*c*) If relevant, consider translating estimates of relative risk into absolute risk for a meaningful time period | | P.19-20, Figure.2,3, Table. S3-7 | |
| Other analyses | 17 | Report other analyses done—eg analyses of subgroups and interactions, and sensitivity analyses | | P.20,21  Figure.4 Table. S8-13  Figure. S2-3 | |
| Discussion | | | |  | |
| Key results | 18 | Summarise key results with reference to study objectives | | P.22-25 | |
| Limitations | 19 | Discuss limitations of the study, taking into account sources of potential bias or imprecision. Discuss both direction and magnitude of any potential bias | | P.25-27 | |
| Interpretation | 20 | Give a cautious overall interpretation of results considering objectives, limitations, multiplicity of analyses, results from similar studies, and other relevant evidence | | p.27-28 | |
| Generalisability | 21 | Discuss the generalisability (external validity) of the study results | | N/A | |
| Other information | | | |  | |
| Funding | 22 | Give the source of funding and the role of the funders for the present study and, if applicable, for the original study on which the present article is based | | P.28 | |

*Give information separately for cases and controls in case-control studies and, if applicable, for exposed and unexposed groups in cohort and cross-sectional studies.

**Note:** An Explanation and Elaboration article discusses each checklist item and gives methodological background and published examples of transparent reporting. The STROBE checklist is best used in conjunction with this article (freely available on the Web sites of PLoS Medicine at http://www.plosmedicine.org/, Annals of Internal Medicine at http://www.annals.org/, and Epidemiology at http://www.epidem.com/). Information on the STROBE Initiative is available at www.strobe-statement.org.
