# Supplementary figures and images for: Exploring the mortality and cardiovascular outcomes with SGLT-2 inhibitors in patients with T2DM at dialysis commencement: a health global federated network analysis
Source: Cardiovasc Diabetol. 2024 Sep 3;23:327. doi: 10.1186/s12933-024-02424-7 (PMC11373240; doi:10.1186/s12933-024-02424-7)

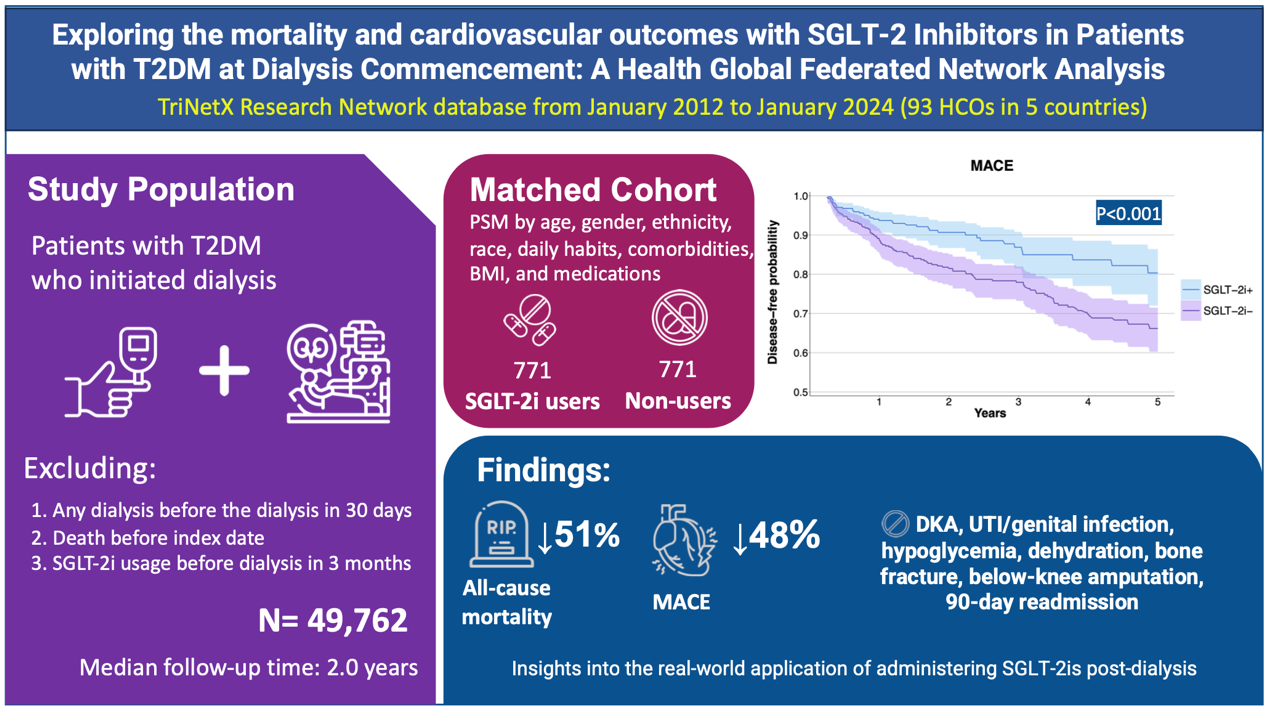

Supplement: Supplementary file 2 — Supplementary Material 2 [file 12933_2024_2424_MOESM2_ESM.docx]
